# Morphological Divergence Without Genetic Differentiation in Widely Distributed Chinese *Cipangopaludina* Species

**DOI:** 10.3390/biology15151317

**Published:** 2026-08-06

**Authors:** Yaozong Wang, An Li, Fuguang Luo, Ziyi Wang, Xiao Chen, Zhiqiang Wang, Qianhong Gu

**Affiliations:** 1 Engineering Research Center of Polyploid Fish Reproduction and Breeding of the State Education, Ministry, College of Life Sciences, Hunan Normal University, Changsha 410081, China; 202330233036@hunnu.edu.cn (Y.W.); 202330233042@hunnu.edu.cn (A.L.); 202130233024@hunnu.edu.cn (Z.W.); chenxiao01s@163.com (X.C.); 2 Key Laboratory of Aquatic Healthy Breeding and Nutrition Regulation of Guangxi Universities, College of Animal Science and Technology, Guangxi University, Nanning 530004, China; luofuguang3563@163.com; 3 Liuzhou Aquaculture Technology Extending Station, Liuzhou 545006, China; wangzhiqiang2019@foxmail.com

**Keywords:** *Cipangopaludina*, geometric morphometrics, phylogenetic analysis, species delimitation, sexual dimorphism

## Abstract

The taxonomic status of *Cipangopaludina chinensis* and *C. cathayensis* remains controversial due to morphological overlap. We combined morphometrics (24 landmarks vs. 200 semilandmarks) with multi-locus phylogenetics (*COI*, *16S rRNA*, *H3*, *28S rRNA*) and haplotype networks on sympatric populations from Liuzhou and GenBank. Morphometrics completely separated *C. chinensis*_lz from *C. cathayensis*_lz in shell shape; key diagnostic traits include aperture size, spire height, and body whorl inflation. The high-density semilandmark method outperformed discrete landmarks in dimensionality reduction and fine-scale resolution, capturing subtle apex and lateral whorl variations. No sexual dimorphism occurred in *C. cathayensis*_lz, with only weak dimorphism in *C. chinensis*_lz. Molecular data were ambiguous: the species tree and mitochondrial gene tree recovered *C. chinensis* and *C. cathayensis* as monophyletic, but the nuclear gene tree showed mixing of *C. chinensis*_lz/*C. cathayensis*_lz with *C. wisseli*, *C. chinensis*, and *C. cathayensis*. Haplotype networks revealed haplotypes sharing between *C. chinensis*_lz and *C. cathayensis*_lz, yet neither population shared haplotype with GenBank sequences of the two nominal species. Morphology strongly supports *C. chinensis*_lz and *C. cathayensis*_lz as distinct species, whereas molecular evidence shows only low-level differentiation and discordant signals between mitochondrial and nuclear markers. We conclude they are likely valid species, yet mitonuclear discordance warrants further genomic investigation. Despite no evidence of microhabitat partitioning, the stable morphological divergence between these sympatric morphospecies supports separate management units to conserve phenotypic diversity and local adaptive potential. This study validates geometric morphometrics as an efficient frontline tool for biodiversity assessment in morphologically diverse yet genetically conserved freshwater snails, and reinforces the need for genome-wide data to resolve species boundaries within the *Cipangopaludina* complex.

## Simple Summary

The taxonomic status of two Chinese river snails, *Cipangopaludina chinensis* and *C. cathayensis*, has long been disputed due to shell variation and intermediate forms. We combined shell shape analyses (traditional measurements and geometric morphometrics) with molecular phylogenetics (mitochondrial and nuclear genes) to test whether they represent distinct species. Shell shape completely separated the two taxa, with diagnostic differences in aperture, spire, apex and umbilicus. Genetic data were ambiguous: mitochondrial DNA supported the separation of the two Liuzhou populations (*C. chinensis*_lz and *C. cathayensis*_lz) from *C. chinensis* and *C. cathayensis*, but nuclear genes showed mixing, suggesting incomplete lineage sorting or limited gene flow. The studied populations shared no haplotypes with GenBank sequences of either nominal species, indicating they form a distinct genetic lineage. We conclude that *C. chinensis* and *C. cathayensis* are likely valid species, but genomic data are needed to resolve their genetic boundaries. This integrative approach provides a robust framework for species delimitation in morphologically challenging freshwater snails and has implications for their conservation and management.

## 1. Introduction

Freshwater gastropods of the family Viviparidae display remarkable morphological diversity in shell form [1,2,3,4,5,6]. This variation has long confounded taxonomic classification, generating persistent systematic instability across the group [2,4,5,7,8,9,10,11]. For example, shell shape overlap has led to repeated taxonomic revisions in Sinotaia [2], and morphologically similar but distantly related lineages have been conflated within Margarya until molecular data revealed polyphyly [9]. Specifically, in East Asia, this challenge is epitomized by two widespread and economically important congeneric morphospecies, *Cipangopaludina chinensis* (Gray, 1834) and *C. cathayensis* (Heude, 1890), collectively called the Chinese river snails [12]. Both species are widely distributed in East Asian freshwater ecosystems, inhabiting lakes, ponds, reservoirs, slow-flowing rivers, and rice paddies with soft, muddy substrates. For over a century, these taxa have been distinguished primarily by shell shape. The key traits used include height-to-width ratio, spire height, body whorl outline, and aperture form. Despite these diagnostic traits, field surveys frequently uncover intermediate forms that resist clear taxonomic assignment. The critical question, therefore, is whether these nominal taxa represent valid, distinct species, or a single species, in which case one name would be synonymized [2,10]. This taxonomic uncertainty carries significant practical consequences, as both species are harvested extensively for human consumption, serve as critical feed sources in aquaculture, accelerate aquatic nutrient cycling, maintain ecosystem stability through filter-feeding that removes suspended particles and algae from the water column, and act as sensitive bioindicators of water quality in shallow lentic ecosystems [13,14,15,16]. Resolving their systematic status is therefore essential for effective fisheries management, ecological monitoring, and conservation planning.

Landmark-based geometric morphometrics (GM) has revolutionized the analysis of organismal shape by enabling rigorous, size-independent quantification of morphological variation via Generalized Procrustes Analysis, which eliminates non-shape confounding factors such as position, orientation, and overall size [17,18]. Its utility in dissecting adaptive variation has been demonstrated across diverse systems, including squamate osteoderms, mosquito wings, and anuran skeletal morphology [19,20,21]. In molluscan research, this approach has been particularly powerful because it preserves the full geometry of shell shape, providing a robust analytical framework for investigating shell evolution, adaptation, and phenotypic diversification [22,23,24,25], and has proven effective in resolving taxonomic debates, delineating cryptic species, quantifying ecophenotypic plasticity, and detecting subtle sexual dimorphism [26,27,28,29]. Viviparid snails are gonochoristic, and sexual dimorphism in shell morphology has been documented in multiple genera, including *Viviparus*, *Filopaludina* (Habe, 1964), and *Margarya* (Nevill, 1877) [30,31,32,33]. In *Viviparus*, for instance, females exhibit significantly larger shell width, higher body whorl, and larger aperture than males, though these differences are age-dependent and primarily manifest after sexual maturity [30,33]. In *Filopaludina martensi martensi* (Frauenfeld, 1864), sexual differences have been observed in shell and operculum size, with females being larger than males [32]. Similarly, females of *Margarya* are generally larger than males in shell body type [31]. These examples illustrate that sexual shell dimorphism in viviparids—when present—typically involves differences in overall size and, to a lesser extent, shell shape. For the *C. chinensis*–*C. cathayensis* species complex, however, a key question remains unaddressed. It is unclear whether the morphological gap between them reflects species-level divergence, or simply sexual dimorphism and intraspecific variation misclassified by traditional taxonomy. This possibility, a potential driver of taxonomic inflation, has not been previously addressed using an integrative framework combining high-resolution geometric morphometrics and multi-locus phylogenetics specifically for these two nominal species.

Alongside these morphological advances, the widespread application of DNA barcoding and multi-locus phylogenetics has revealed pervasive discordance between morphological and genetic differentiation across numerous mollusk groups [34,35]. This pattern can arise from multiple processes. Incomplete lineage sorting (ILS) retains ancestral polymorphisms after speciation [23,36] and often generates mitonuclear discordance, i.e., conflicting phylogenetic signals between mitochondrial and nuclear markers [1,37]. Introgression via hybridization is another cause, occurring when reproductive barriers are incomplete [36,38]. Phenotypic plasticity is distinct: it produces morphological variation without underlying genetic change [1,23]. These mechanisms differ fundamentally: ILS reflects shared ancestry, introgression indicates gene exchange, and plasticity represents environmental induction of phenotypic variation. In our system, ILS or historical introgression are more plausible explanations than plasticity, given the consistent and sympatric shell differentiation observed [2,24]. Distinguishing among these possibilities, however, will require genome-wide data [36,37]. Preliminary genetic data (based on mitochondrial *COI* sequences) indicate remarkably low interspecific distances between *C. chinensis* and *C. cathayensis* [11,12], yet no integrative study has combined high-resolution geometric morphometrics with multi-locus genetics to resolve this systematic conundrum.

Both species are economically and ecologically important [13,14,15,16]. Resolving their systematic status is therefore essential for effective fisheries management, ecological monitoring, and conservation planning. In this study, we tested two competing hypotheses: (1) *C. chinensis* and *C. cathayensis* represent distinct, independently evolving species, as predicted by their diagnostic shell characters; (2) the observed morphological gap reflects intraspecific variation or sexual dimorphism rather than species-level divergence. To test these hypotheses, we performed the first integrative taxonomic assessment combining landmark-based geometric morphometrics with phylogenetic and haplotype network analyses of four markers—mitochondrial COI and *16S rRNA*, and nuclear *28S rRNA* and H3. These markers represent standard DNA barcoding and phylogenetic loci for gastropods [39,40,41], ensuring comparability with GenBank data. We further included diverse gastropod taxa and outgroups for robust phylogenetic inference. Our findings have implications for the taxonomy, evolution, and conservation of freshwater Viviparidae.

## 2. Materials and Methods

### 2.1. Sample Collection, Traditional and Geometric Morphometrics Analyses

*Cipangopaludina chinensis*_lz and *C. cathayensis*_lz were both collected from one pond (N 24.47°, E 109.07°) in Liuzhou City, Guangxi Zhuang Autonomous Region, with 31 individuals of each species sampled (16 males and 15 females). Species identity was initially assigned in the field based on diagnostic shell characters following Liu et al. [42]: *C. chinensis* was recognized by its slender, high-spired, elongate-conical shell with spire height exceeding aperture height; *C. cathayensis* was identified by its broadly conical to ovoid shell with a short, broad spire and a more inflated body whorl. This a priori classification served as the working hypothesis, which was subsequently tested using traditional and geometric morphometrics, as well as molecular phylogenetic analyses. In addition, all available *Cipangopaludina* sequences for the four markers were retrieved from GenBank, along with other molluscan outgroups (Table S1). Selection was based on sequence completeness and unambiguous genus-level assignment. No geographic filtering was applied, as most records lacked provenance data; this inclusive strategy was adopted to maximize taxonomic coverage for evaluating the phylogenetic position of the Liuzhou populations. To test for morphometric differences between *C. chinensis*_lz and *C. cathayensis*_lz and to assess intraspecific sexual dimorphism, traditional linear measurements were performed on the same specimens as used for geometric morphometric analyses. Seven shell characters—shell height (SH), shell width (SW), spire height (SpH), body whorl height (BWH), aperture width (AW), operculum height (OH), and operculum width (OW)—were measured using digital calipers (0.01 mm precision, Table S2). To preliminarily assess shape variation prior to formal morphometric analyses, all raw measurements were divided by shell height (SH) to obtain morphometric ratio indices (SW/SH, SpH/SH, BWH/SH, AW/SH, OH/SH, OW/SH). This ratio approach was adopted because all specimens were adults with limited size variation—shell height ranged from 51.84 to 60.88 mm in *C. chinensis*_lz and from 43.06 to 58.21 mm in *C. cathayensis*_lz (Table S2)—rendering allometric effects negligible. Principal component analysis (PCA) based on the covariance matrix and principal coordinate analysis (PCoA) based on Euclidean distance matrices were conducted on the ratio data, followed by pairwise Wilcoxon rank-sum tests (wilcox.test) with Bonferroni correction (α = 0.05). All statistical analyses were performed in R 4.2.1. Prior to ANOVA, the assumptions of normality and homogeneity of variances were assessed for each of the six proportional traits. Shapiro–Wilk tests confirmed normality of residuals (all *p* > 0.05) and Levene’s tests confirmed homogeneity of variances (all *p* > 0.05), indicating that the data met the requirements for parametric analysis.

Furthermore, shell geometric morphometrics was also used to examine shape variation in MorphoJ v. 2.0 [43]. Two complementary landmark-based geometric morphometric approaches were employed to quantify shell shape variation. Each specimen was assigned a unique identification number and photographed in a standardized orientation and at a fixed focal length using a Canon EOS R50 digital camera, ensuring image consistency across all samples. The first method followed the protocol of Minton & Wang [31], who demonstrated sexual shape dimorphism in *Viviparus* (Gastropoda: Viviparidae) (Montfort, 1810) using a set of 24 type II landmarks (homology inferred based solely on relative positional correspondence) placed at suture lines or extreme points on the shell surface (as illustrated in Supplemental Figure S1). These landmarks were digitized on each shell image using tpsDig2 [44]. The X and Y coordinates of all landmarks were recorded and subjected to Generalized Procrustes Analysis (GPA) in tpsRelw [44] to remove non-shape variation arising from differences in position, orientation, and scale. The resulting Procrustes coordinates were imported into MorphoJ v2.0 for subsequent multivariate analyses. The second approach employed high-density outline-based semilandmarks to capture fine-scale shell contour variation. Shell images were compiled into a single TPS file using tpsUtil [44]. In tpsDig2, the “draw curves” function was used to trace each shell outline counterclockwise from the apex around the entire shell perimeter. Each curve was then resampled into 200 equidistant semilandmarks using the “resample curves” function with the “by length” option. This method generates points that are geometrically, rather than anatomically, corresponding across specimens, and thus represents a complementary strategy for shape quantification. Because MorphoJ does not recognize curve control lines (“LM = 0, CURVES = 1, POINT = 150/200/250”), the file was opened in a text editor and these lines were batch-replaced with “LM = 200” prior to import, retaining 200 equidistant semilandmarks for analysis.

For both landmark datasets, subsequent analyses followed the same workflow in MorphoJ v2.0. Datasets for each species were imported separately and then merged using “Preliminaries → Combine Datasets.” Outliers were identified and removed using the “Find Outliers” function based on the 95th percentile of Procrustes distances (default threshold). Covariance matrices were generated via “Preliminaries → Generate Covariance Matrix.” Principal component analysis (PCA) was performed on both single-species and combined datasets to visualize patterns of shape variation. For the combined dataset, a classifier variable was created with four categories: *C. chinensis*_lz female (ZGC, n1 = 15), *C. chinensis*_lz male (ZGX, n2 = 16), *C. cathayensis*_lz female (ZHC, n3 = 15), and *C. cathayensis*_lz male (ZHX, n4 = 16). Canonical variate analysis (CVA) was conducted using both species identity and the combined species–sex classifier to maximize discrimination among predefined groups. To assess the classification power of the CVA and guard against potential overfitting due to the high-dimensional nature of the shape data relative to sample size, leave-one-out cross-validation (jackknife) was performed [45]. In this procedure, the CVA function was repeatedly recalculated using N-1 individuals and used to classify the single omitted individual, with the process iterated for all N individuals. To evaluate the statistical significance of group differences, pairwise permutation tests (10,000 iterations) were performed. Pillai’s trace test was not used, as it relies on the inversion of the within-group covariance matrix, which becomes singular when the number of shape variables (*p*) exceeds the sample size (n). Under such conditions, the test statistic cannot be reliably estimated. Instead, group comparisons were based on Goodall’s F test, which is derived from Procrustes distances and does not require matrix inversion, and permutation tests, which are distribution-free and robust to small sample sizes. Both approaches are statistically robust and appropriate for the present dataset. For both PCA and CVA, results including transformation grids, scatter plots with confidence ellipses, and Procrustes and Mahalanobis distances with associated *p*-values were exported.

### 2.2. DNA Extraction, Sequencing and Molecular Analyses

Foot muscle tissue was used for genomic DNA extraction with a QIAGEN DNeasy Blood & Tissue Kit (Shanghai, China) following the manufacturer’s instructions. DNA concentration and purity were assessed using a NanoDrop 2000 spectrophotometer (Thermo Fisher Scientific, USA). Four molecular markers—two mitochondrial (*COI* and *16S rRNA*) and two nuclear (*H3* and *28S rRNA*)—were targeted for PCR amplification. Primers for *COI* (LCO1490/HC02198), *16S rRNA* (16sar-L/16sbr-H), and *H3* (F/R) were obtained from published sources [39,40,41]. For *28S rRNA* gene amplification, we referred to Gu et al. (2019) [23] but introduced minor modifications to the PCR protocol to ensure robust amplification of this locus. Amplification was performed in 50-μL reactions containing 1 μL of DNA template, 10 μL of 5× PrimeSTAR Buffer (Mg^2+^ plus), 4 μL of dNTP mixture (2.5 mM each), 1 μL of each primer (all primers were used at a final concentration of 10 µM in each 50 µL PCR reaction), 0.5 μL of PrimeSTAR HS DNA Polymerase (2.5 U/μL; TaKaRa, Dalian, China), and sterile water to volume. Thermal cycling consisted of an initial denaturation at 94 °C for 4 min, followed by 35 cycles of 98 °C for 10 s, annealing for 5 s, and extension at 72 °C for 45 s, with a final 8 min extension at 72 °C. Annealing temperatures differed among loci: 50 °C for *COI*, 48 °C for *16S rRNA*, 54 °C for *H3*, and 52 °C for *28S rRNA*.

PCR products were purified with a commercial purification kit and sequenced bidirectionally using BigDye Terminator chemistry on an automated sequencer. Sequence reads were aligned using MAFFT as implemented in PhyloSuite v1.2.3 [46], and checked against GenBank entries for verification. All newly obtained sequences have been deposited in GenBank; accession numbers are provided in Table S1.

### 2.3. Phylogenetic Analyses

To evaluate the hypothesis that the nominal *Cipangopaludina* species under study constitute monophyletic lineages, species-tree inference was performed using the multi-locus coalescent method implemented in BEAST v2.6.7 [47]. The analysis encompassed 32 individuals for which sequences from all four targeted markers were available (Table S1). Five species (*Gigantopelta aegis* Chen et al., 2015, *Chrysomallon squamiferum* Chen et al., 2015, *Gibbula magus* Linnaeus, 1758, *Steromphala cineraria* Linnaeus, 1758, *Phorcus lineatus* da Costa, 1778) were designated as outgroups. Optimal nucleotide substitution models were selected independently for each gene partition using PartitionFinder as implemented in PhyloSuite v1.2.3. The resulting assignments were as follows: GTR + F + I + G4 for *COI* and *H3*, GTR + F + I + G4 for *28S rRNA*, and GTR + F + I + G4 for *16S rRNA*. Prior to multi-locus analysis, a partition homogeneity test (implemented as the incongruence length difference test) was conducted in PAUP* (Phylogenetic Analysis Using Parsimony *and Other Methods, version 4.0b10) to assess combinability of the four gene fragments [48]. The test yielded a *p* > 0.01, indicating no significant phylogenetic conflict among the partitions and supporting their combined analysis. Input files for the coalescent analysis were generated with BEAUTi v2 available in the BEAST package. A Yule pure-birth process was specified as the species-tree prior, consistent with recommendations for species-level datasets [49], and a constant population size was assumed. The Markov chain Monte Carlo (MCMC) chain was run for 30 million generations, with parameters and trees sampled at intervals of 3000 generations. Adequate mixing and convergence were verified in Tracer v1.7 [50] by confirming that effective sample sizes (ESS) for all parameters exceeded 200. The initial 10% of sampled trees (1000 trees) were discarded as burn-in after visual inspection confirmed that likelihood values had reached a stationary plateau. A maximum-clade-credibility consensus tree was subsequently summarized using TreeAnnotator v2.6.7 available in the BEAST package, again with 10% burn-in removed, and nodes receiving posterior probability support ≥ 95% were considered strongly supported. The resulting species tree was visualized in FigTree v1.4.4 (https://tree.bio.ed.ac.uk/software/figtree/, accessed on 25 May 2026). To further assess topological concordance across the posterior sample, all post-burn-in trees were superimposed as transparent lines using DensiTree v2.01 [51]; regions of the resulting plot exhibiting dense coloration indicate high agreement among trees on a particular topology and set of branch lengths.

Phylogenetic relationships were also reconstructed separately for mitochondrial (*COI* + *16S rRNA*) and nuclear (*H3* + *28S rRNA*) concatenated datasets using maximum likelihood (ML) and Bayesian inference (BI). The concatenated analyses used a broader sample than the species-tree analysis: both the mitochondrial and nuclear datasets included all successfully sequenced individuals plus GenBank sequences (*n* = 81, Table S1). All *C. chinensis*_lz and *C. cathayensis*_lz individuals were included in both datasets. The 32 individuals used in the species-tree analysis (with all four markers) are a subset of these larger datasets (Table S1). All sequences were aligned with MAFFT in PhyloSuite v1.2.3. Protein-coding genes (*COI* and *H3*) were partitioned by codon position; ribosomal genes (*16S rRNA* and *28S rRNA*) were treated as single partitions. The optimal nucleotide substitution model for each partition was selected with ModelFinder, with separate runs conducted for the mitochondrial and nuclear datasets. Base saturation was assessed under the GTR model in DAMBE v7.3.11 (https://dambe.bio.uottawa.ca/DAMBE/dambe.aspx, accessed on 21 March 2026). Maximum likelihood trees for each dataset were inferred with RAxML 8.0 [52], applying the GTR + G model to all partitions and performing 1000 bootstrap replicates to assess nodal support. Bayesian analyses were run in MrBayes 3.2.2 [53], with each dataset partitioned according to the optimal schemes identified by ModelFinder. Two independent MCMC runs, each with four chains, were executed for approximately 10 million generations, sampling every 1000 generations. Convergence was evaluated in Tracer v1.7 by verifying that the average standard deviation of split frequencies fell below 0.01 and that effective sample sizes (ESS) exceeded 200 for all parameters. The initial 10% of sampled trees were discarded as burn-in. Final consensus trees were visualized and edited in iTOL (https://itol.embl.de/, accessed on 18 May 2026). To detect potential phylogenetic conflicts between mitochondrial and nuclear markers, the topologies of the resulting ML and BI trees from the two datasets were compared visually in FigTree, focusing on well-supported nodes. Well-supported nodes (bootstrap ≥ 70% in ML; posterior probability ≥ 0.95 in BI) that exhibited discordant relationships between the mitochondrial and nuclear gene trees were identified and recorded.

Furthermore, we generated the haplotype network for the *COI* data (*n* = 244) and *16S rRNA* (*n* = 235, including all *Cipangopaludina* samples, Table S3) and colored each haplotype by species. Median-joining haplotype networks for *COI* and *16S rRNA* were constructed in PopART 1.7 [54,55].

## 3. Results

### 3.1. Morphological and Sexual Divergence

#### 3.1.1. Traditional Morphometrics

**Figure 1:**
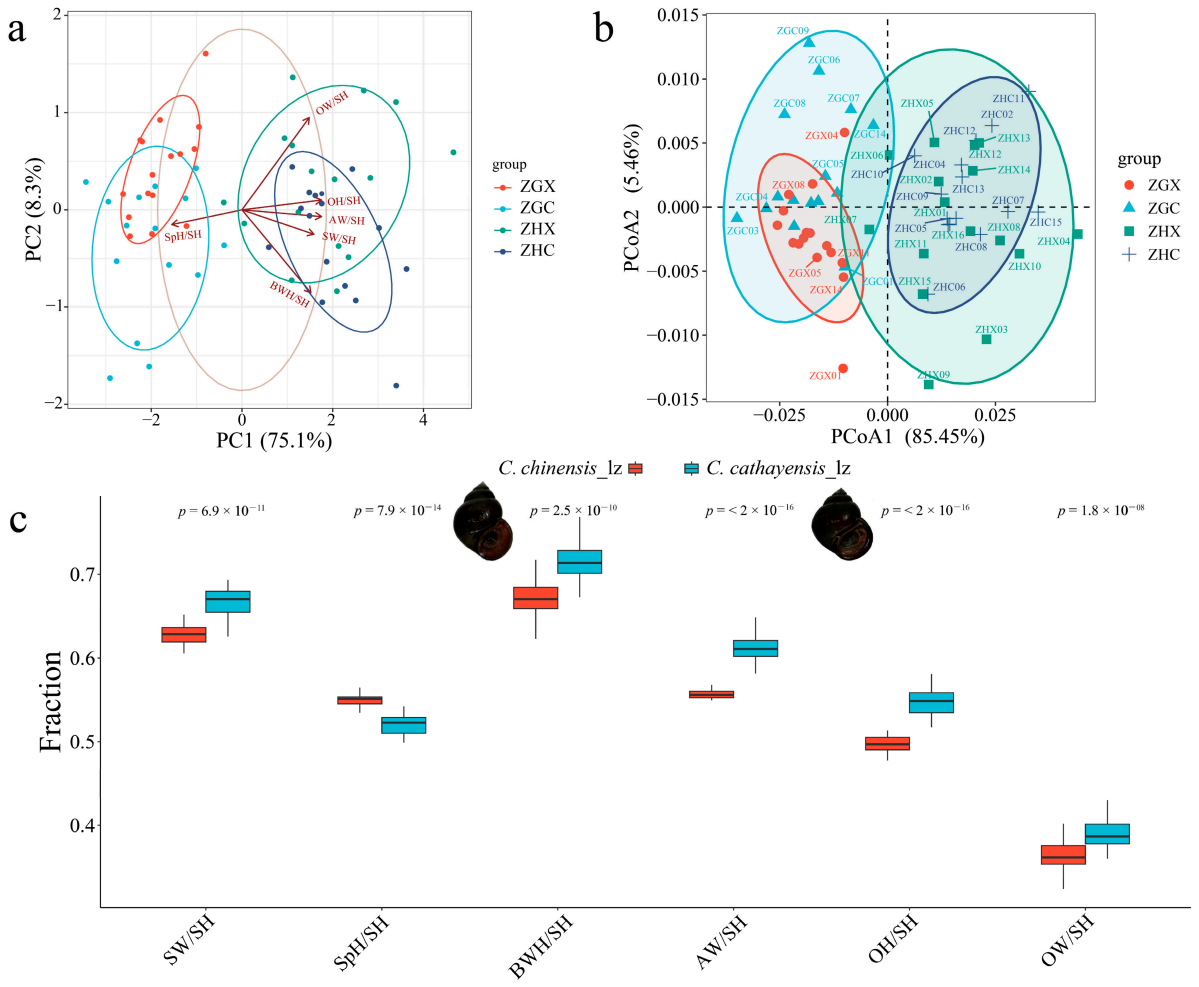
(**a**) Principal component analysis (PCA) based on the covariance matrix of proportional morphological measurement (Table S2), showing separation between *C. chinensis*_lz and *C. cathayensis*_lz along PC1 (68% confidence ellipses), and (**b**) Principal coordinates analysis (PCoA) based on morphological distance matrices, with PCoA1 and PCoA2 together accounting for 90.91% of total variation. Both analyses are used descriptively to visualize multivariate morphological patterns; no formal hypothesis testing is associated with these ordinations, and (**c**) the statistical significance of intergroup differences was evaluated by pairwise Wilcoxon rank-sum tests (wilcox.test). Specimens are grouped by taxon and sex: ZGX denotes male individuals of *C. chinensis*_lz, ZGC denotes female individuals of *C. chinensis*_lz, ZHX denotes male individuals of the *C. cathayensis*_lz taxon, and ZHC denotes female individuals of *C. cathayensis*_lz; Arrows in the plot correspond to the loading vectors of the input shape ratio indicators: the orientation of each arrow reflects the direction of correlation between the respective morphological trait and the two principal component axes, indicating how the trait value shifts along the PC gradients; arrow length is proportional to the trait’s contribution to the total variance captured by PC1 and PC2, with longer arrows indicating a stronger driving effect on the observed morphological differentiation among groups.

PCA based on the covariance matrix showed that PC1 and PC2 explained 75.1% and 8.3% of total variation, respectively. *Cipangopaludina chinensis*_lz and *C. cathayensis*_lz were completely separated along PC1 (68% confidence ellipses non-overlapping), indicating significant shell differentiation (Figure 1a). No sex-associated shape variation was detected within either taxon. Loading analysis identified SpH/SH (negative) and AW/SH, SW/SH, OW/SH, OH/SH, BWH/SH (positive) as major contributors to PC1, while OW/SH and BWH/SH also contributed to PC2, reflecting within-group variation (Figure 1a). PCoA based on morphological distance matrices showed that PCoA1 and PCoA2 explained 85.45% and 5.46% of variation (cumulative 90.91%, Figure 1b), with complete separation of the two taxa along PCoA1 and sexes interspersed within each group. Shapiro–Wilk and Levene’s tests confirmed normality and variance homogeneity for all six traits (all *p* > 0.30). Two-way ANOVA followed by Tukey’s HSD revealed highly significant interspecific differences for all six traits (SW/SH, SpH/SH, BWH/SH, AW/SH, OH/SH, OW/SH; all *p* < 0.001, Figure 1c), reinforcing the pronounced shell differentiation between the two taxa.

#### 3.1.2. Geometric Morphometrics

**Figure 2:**
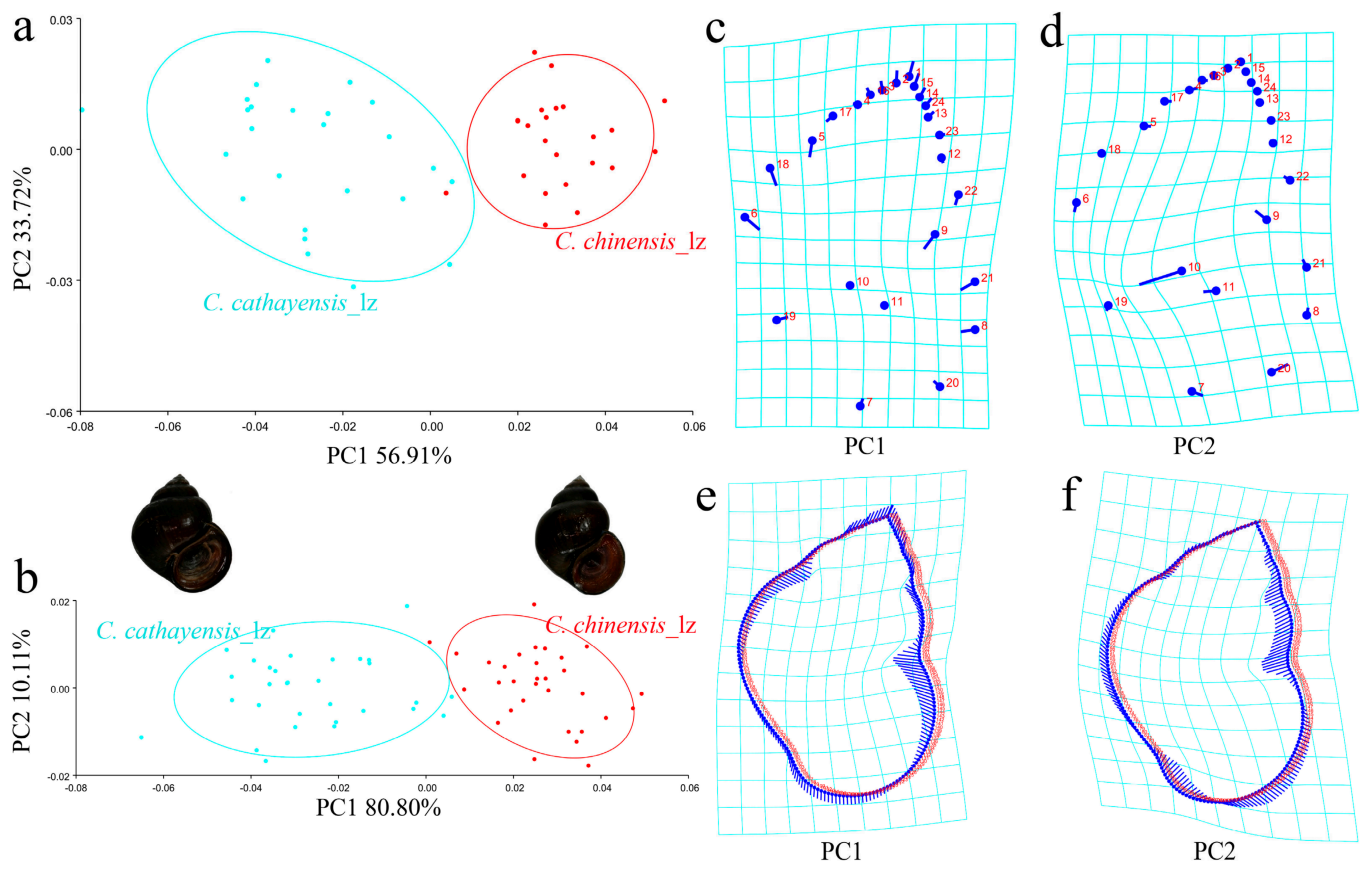
Results of principal component analysis (PCA) for two groups *C. cathayensis*_lz and *C. chinensis*_lz. (**a**,**b**) PCA scatter plots constructed from 24 landmarks and 200 dense semilandmarks, respectively; (**c**,**d**) Morphological deformation grids of PC1 and PC2 based on 24 landmarks; (**e**,**f**) Morphological deformation grids of PC1 and PC2 based on 200 dense semilandmarks.

Two geometric morphometric approaches (24 discrete landmarks vs. 200 high-density semilandmarks) were applied. PCA showed that the first nine principal components from discrete landmarks accounted for 90.63% of total shape variation (PC1 alone contributed 56.91%) (Figure 2a), while the first three components from high-density semilandmarks captured 90.91% of variation (PC1 alone contributed 80.80%) (Figure 2b), demonstrating superior dimensionality reduction efficiency. Both methods clearly separated the two species along PC1: *C. cathayensis*_lz clustered on the negative side and *C. chinensis*_lz on the positive side, with the latter exhibiting a more compact distribution and minimal individual overlap (Figure 2). Thin-plate spline deformation grids identified core interspecific differences in the outer aperture lip, left side of the first whorl near the aperture, and umbilicus: *C. cathayensis*_lz had an expanded aperture and adjacent whorl, a depressed apex, and a flatter overall shell, whereas *C. chinensis*_lz showed a retracted aperture and whorl, an elongated apex, and a more slender form (Figure 2c,d). The high-density semilandmark method further resolved subtle apex variation and contraction/expansion of the right whorl, significantly improving morphological resolution (Figure 2e,f).

**Figure 3:**
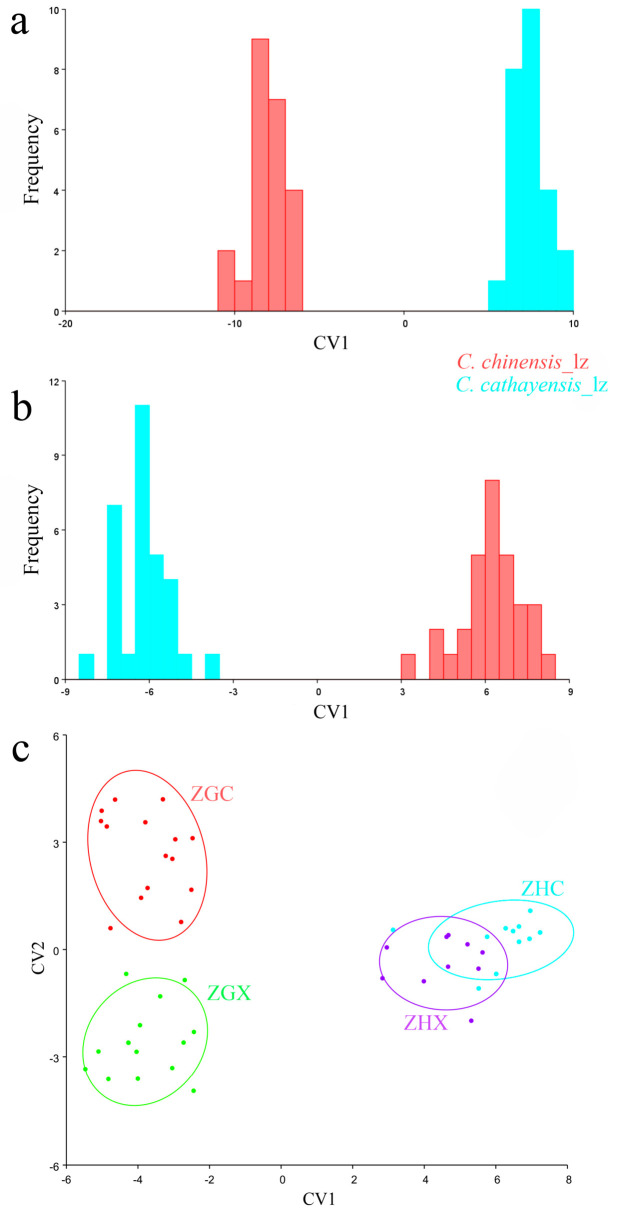
Results of canonical variate analysis (CVA) for shell morphological variation. (**a**,**b**) Frequency histograms of CV1 scores comparing populations *C. cathayensis*_lz and *C. chinensis*_lz, constructed using 24 landmarks and 200 dense semilandmarks, respectively; (**c**) Two-dimensional CVA scatter plot along CV1 and CV2 axes based on 200 dense semilandmarks, illustrating morphological differentiation among four subgroups delineated by sampling population and sex (ZGC, ZGX, ZHC, ZHX).

Leave-one-out cross-validation confirmed the robustness of the CVA classification, with 98.4% of individuals correctly assigned to their a priori groups (compared to 100.0% in the original non-cross-validated classification). CVA revealed that CV1 explained 100% of interspecific variation for both datasets, with complete separation of the two groups along this axis (Figure 3a,b). Interspecific Mahalanobis distances were 15.4901 (discrete landmarks) and 12.3682 (high-density semilandmarks), with corresponding Procrustes distances of 0.0573 and 0.0524, respectively (Table 1). All 10,000-iteration permutation tests and Goodall’s *F* tests confirmed highly significant interspecific differences (*p* < 0.0001). For species-sex grouped analysis, the first two CVA axes from high-density data explained 95.56% of among-group variation. Interspecific Mahalanobis distances (12.0114–12.7506) were substantially larger than intraspecific sexual distances (3.8391–4.0049) (Table 2). In contrast, the 24-landmark dataset yielded consistently high Mahalanobis distances across all pairwise group comparisons (interspecific: 20.9306–71.8304; intraspecific sexual: 25.2567–35.7758), with no clear separation between interspecific and intraspecific sexual distances. Permutation tests revealed significant intergroup differences (*p* < 0.0001); weak but significant sexual dimorphism was detected only in *C. chinensis*_lz (*p* = 0.0078) using high-density data (Figure 3c), whereas no significant dimorphism was observed in *C. cathayensis*_lz (*p* = 0.2740). Discrete landmark data showed similar trends, but intraspecific sexual differences were not statistically significant.

### 3.2. Phylogenetic Analysis

#### 3.2.1. Species Tree

**Figure 4:**
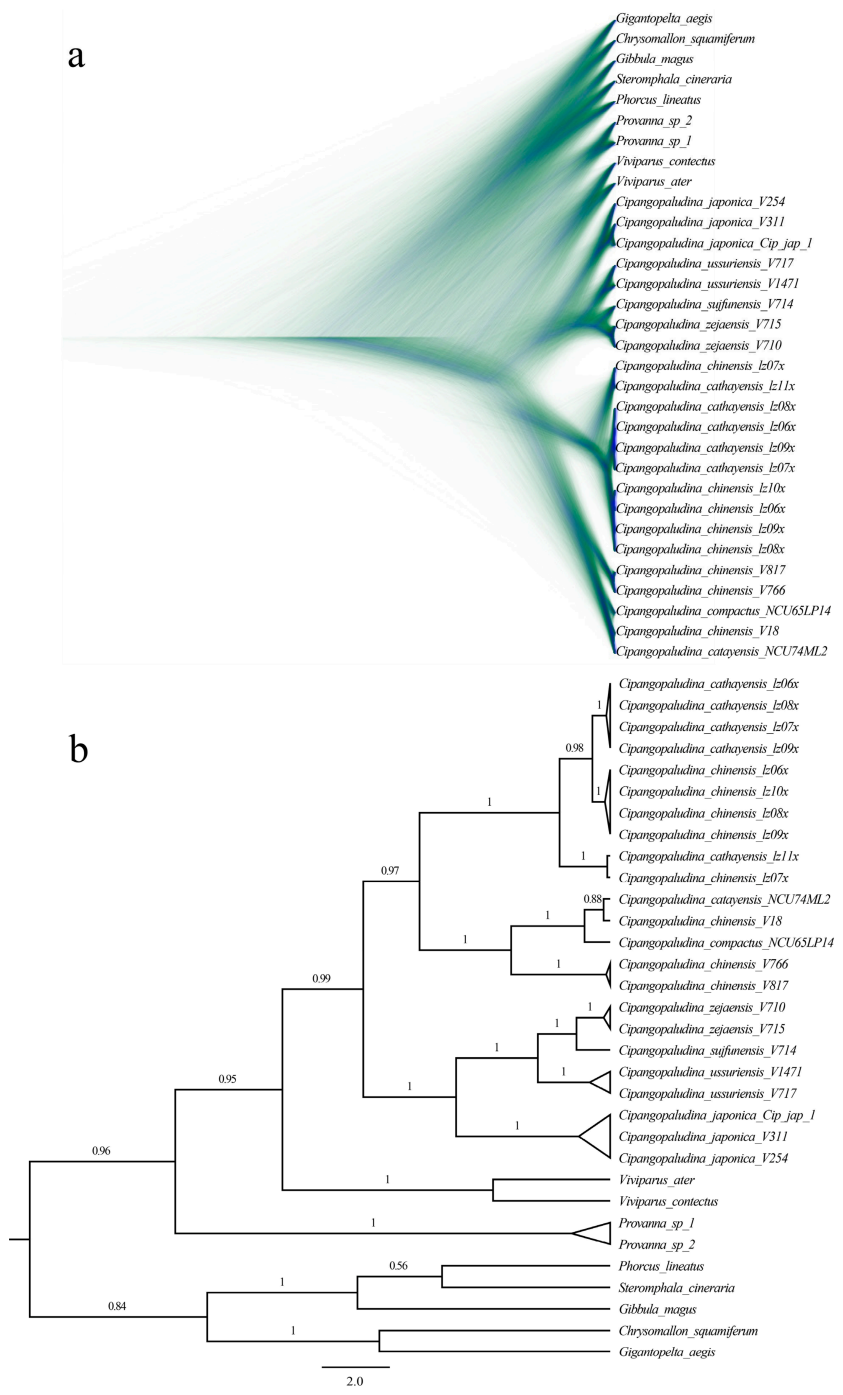
BEAST2-reconstructed species trees to resolve interspecific phylogenetic relationships within the genus *Cipangopaludina*. The phylogenetic inference was built on the concatenated dataset consisting of mitochondrial *COI*, *16S rRNA* and nuclear *H3*, *28S rRNA* gene sequences, and other gastropods, bivalves and cephalopods were included as outgroups. (**a**) Full collection of posterior trees visualized in DensiTree; (**b**) Final summarized species tree generated by TreeAnnotator, with posterior probability values labeled on each evolutionary clade.

The species tree was reconstructed for the genus *Cipangopaludina* using a multispecies coalescent model in BEAST2, based on 32 individual sequences from these four molecular markers (Figure 4a). The outgroup, including *G. aegis*, *C. squamiferum*, *G. magus*, *S. cineraria*, *P. lineatus*, was positioned at the base of the tree. Overlay of the full posterior tree set in DensiTree showed substantial overlap of the major branches, indicating a robust backbone topology. In the phylogenetic tree, *Cipangopaludina* was recovered as a strongly supported monophyletic group (PP = 0.99). All seven nominal species included in this study—*C. japonica*, *C. tussurientis*, *C. suffiensis*, *C. ziaenensis*, *C. chinensis*, *C. compactus*, and *C. cathayensis*—formed distinct monophyletic lineages. Most intra- and interspecific divergence nodes had PP ≥ 0.95 or 1.0. The basal node of the *C. chinensis* complex (including its close relatives) also received high support (Figure 4b). All field-collected samples of the *C. chinensis*_lz and *C. cathayensis*_lz series fell within the *C. chinensis* complex. DensiTree overlay revealed extensive branch overlap and blurred lineage boundaries between *C. chinensis*_lz and *C. cathayensis*_lz, indicating an absence of significant genetic differentiation and a failure to form reciprocally monophyletic evolutionary lineages. Nevertheless, both *C. chinensis*_lz and *C. cathayensis*_lz exhibited clear genetic differentiation from the seven nominal species of *Cipangopaludina*, especially from *C. chinensis* and *C. cathayensis* (Figure 4a).

#### 3.2.2. Gene Tree

**Figure 5:**
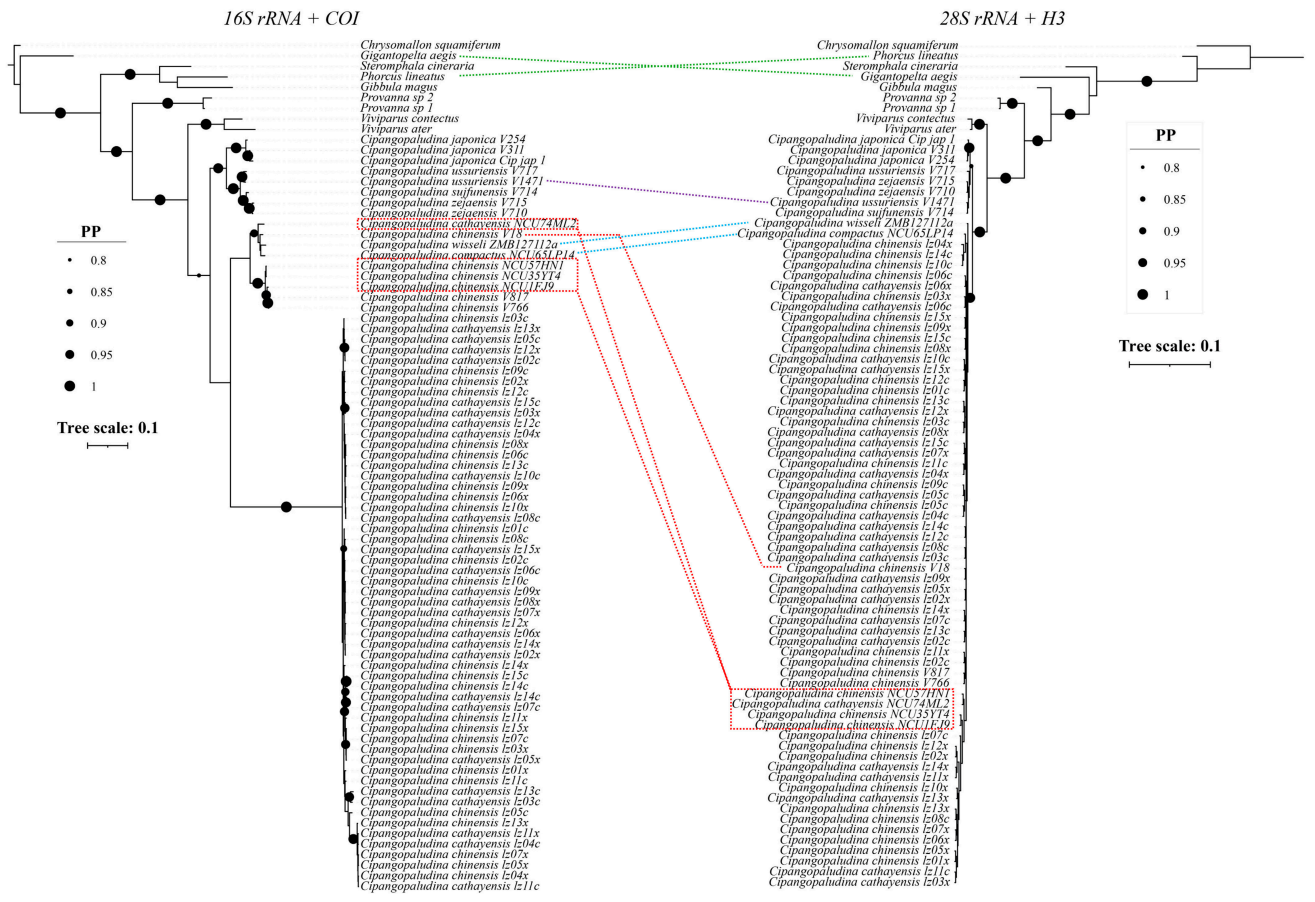
Gene trees inferred from mitochondrial (*COI* + *16S rRNA*) and nuclear (*28S rRNA* + *H3*) genes. The datasets include all samples of ZGC/ZGX and ZHC/ZHX, other taxa of the genus *Cipangopaludina*, and additional gastropods sequences retrieved from GenBank (Table S1). Obvious mitonuclear conflicts were observed in both outgroup placement and interspecific relationships within *Cipangopaludina* between the mitochondrial and nuclear gene trees (indicated by dashed lines connecting conflicting nodes; different colors represent different taxonomic groups).

Separate maximum likelihood (ML) and Bayesian inference (BI) analyses of concatenated mitochondrial (*COI* + *16S rRNA*) and nuclear (*H3* + *28S rRNA*) datasets produced congruent topologies. Mitochondrial trees showed generally higher nodal support than nuclear trees, which exhibited overall low support (Figure 5). Specifically, within *Cipangopaludina*, the vast majority of nodes in the nuclear tree fell below the conventional support thresholds (bootstrap < 70%; PP < 0.95). Significant mitonuclear phylogenetic conflicts were detected outside Viviparidae, as exemplified by the reversed relative positions of the *P. lineatus* and *G. aegis* clades between mitochondrial and nuclear gene trees (Figure 5).

Within *Cipangopaludina*, the mitochondrial phylogenetic tree resolved all seven nominal species as monophyletic groups. *Cipangopaludina chinensis* and *C. cathayensis* were identified as sister taxa, with *C. compactus* forming a clade that included them. The *C. chinensis*_lz and *C. cathayensis*_lz samples were nested within the *C. chinensis* complex, exhibiting differentiation from both *C. chinensis* and *C. cathayensis* (Figure 5). However, these samples displayed short branch lengths and blurred lineage boundaries among each other. The nuclear tree supported the monophyly of *Cipangopaludina* and the independence of the seven nominal species, but conflicted with the mitochondrial tree regarding *C. chinensis*_lz/*C. cathayensis*_lz. These samples did not form a distinct lineage but instead intermixed with individuals of *C. chinensis*, *C. cathayensis*, and *C. wisseli*, exhibiting paraphyly or polytomies.

### 3.3. Genetic Diversity and Haplotype Network

Genetic diversity indices for the focal populations are summarized in Table S3. Mitochondrial markers showed substantially higher variation than nuclear markers. For *COI*, 17 haplotypes were recovered (Hd = 0.933 ± 0.029; π = 0.0139 ± 0.0025), with *C. chinensis*_lz displaying slightly higher diversity than *C. cathayensis*_lz. For *16S rRNA*, five haplotypes were detected (Hd = 0.672 ± 0.059; π = 0.0081 ± 0.0022), with both species showing comparable diversity. In contrast, nuclear markers exhibited limited variation: *H3* yielded five haplotypes (Hd = 0.608 ± 0.007; π = 0.0030 ± 0.0006), whereas *28S rRNA* was completely invariable (Hd = 0.000 ± 0.000).

**Figure 6:**
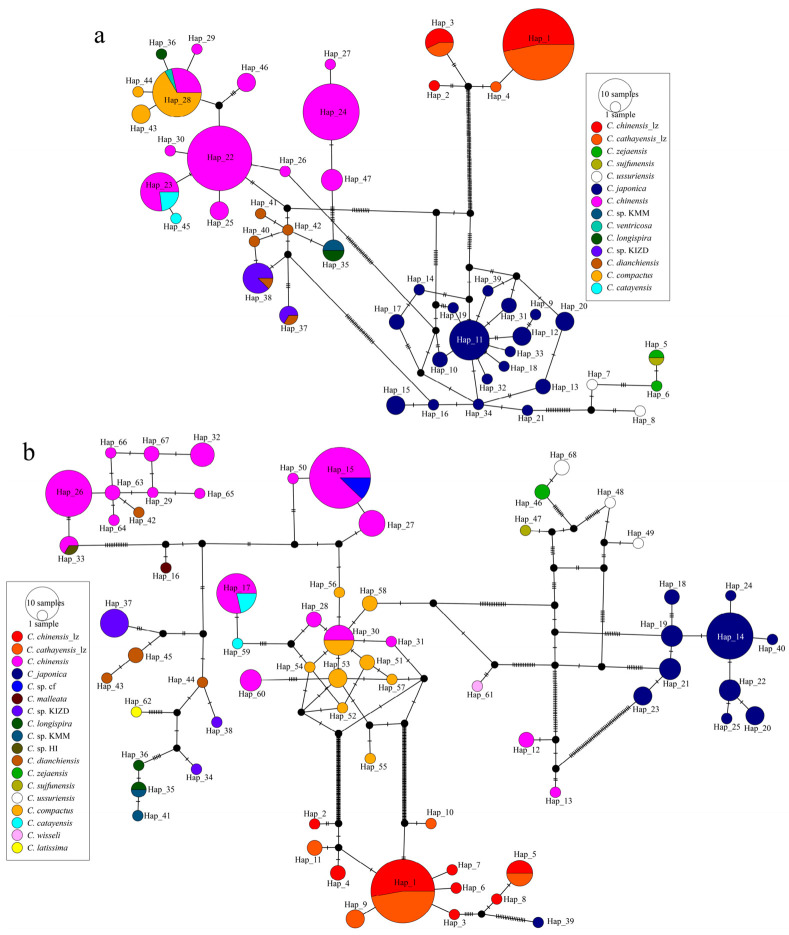
Haplotype networks constructed using *16S rRNA* (**a**) and *COI* (**b**) gene sequences of *Cipangopaludina* retrieved from GenBank (Table S4), together with all samples of *C. chinensis*_lz and *C. cathayensis*_lz. Conspecific species of the genus *Cipangopaludina* are marked with unified colors. The circle size corresponds to haplotype frequency; each connecting line indicates a single nucleotide substitution, and each small tick represents a mutational position.

Median-joining haplotype network analyses based on the *16S rRNA* and *COI* genes yielded overall consistent results. The focal populations *C. chinensis*_lz and *C. cathayensis*_lz formed distinct, independent haplotype lineages (Figure 6a,b). In the *16S rRNA* dataset, four haplotypes (Hap_1–4) were detected with sharing between the two populations; in the *COI* dataset, the two populations shared a single haplotype (Hap_1). No haplotype sharing was observed between these focal populations and the GenBank sequences of *C. chinensis* and *C. cathayensis*; they were separated by many mutational steps, indicating clear lineage segregation. Overall, interspecific genetic boundaries were clearly defined among most congeneric species. Haplotype sharing was restricted to the *C. chinensis* species complex (encompassing *C. cathayensis* and *C. compactus*) and between *C. zejaensis* and *C. sujfunensis*, suggestive of genetic introgression or ambiguous taxonomic delimitation. The vast majority of remaining congeneric taxa formed discrete haplotype clusters in both genes, with substantial interspecific genetic distances and unambiguous species differentiation. Several undetermined specimens shared haplotypes with their corresponding known species and exhibited high genetic affinity, presumably representing misidentified specimens or recently diverged lineages.

## 4. Discussion

This integrative study provides the first simultaneous assessment of shell morphology and multi-locus genetic data for two sympatric Chinese river snail populations, *C. chinensis*_lz and *C. cathayensis*_lz (morphospecies traditionally assigned to *Cipangopaludina* chinensis and *C. cathayensis*, respectively). Our results reveal a pronounced mitonuclear discordance: morphometric analyses consistently and unambiguously separate the two taxa at the interspecific level, whereas molecular data exhibit only modest and partially conflicting signals of genetic divergence. This incongruence may reflect genuine evolutionary processes, such as incomplete lineage sorting or historical introgression, but could also be partially attributable to the limited phylogenetic informativeness of the nuclear markers (*H3* and *28S rRNA*) employed. Resolving the relative contributions of these factors will require genome-wide data. Regardless, the observed mitonuclear discordance reinforces the necessity of integrative taxonomy for species delimitation in morphologically labile freshwater gastropods, and underscores the independent diagnostic value of high-resolution morphometric data when molecular evidence yields equivocal signals.

### 4.1. Morphological Evidence: Clear and Consistent Species Separation

Three multi-scale morphometric approaches all recovered clear shell shape divergence between *C. chinensis*_lz and *C. cathayensis*_lz. They ranged from traditional linear measurements and 24-landmarks geometric morphometrics to 200-point high-density semilandmark outline analysis, and differed substantially in resolving power and trait detection capacity. Traditional linear measurements provided size-adjusted shape discrimination. The complete separation along PC1 and PCoA1 demonstrated that overall shell proportions differ profoundly between the two taxa (Figure 1). The proportional traits driving this separation (SpH/SH, AW/SH, SW/SH, OW/SH, OH/SH, BWH/SH) directly correspond to the classic diagnostic characters described for *C. chinensis* and *C. cathayensis* (Figure 1). The morphological descriptions of *C. chinensis* and *C. cathayensis* follow Liu et al. [42], the standard taxonomic reference for Chinese *Cipangopaludina* [30]. However, Liu et al. [42] is a regional faunal manual rather than a revision based on topotypical material, and the original descriptions date back to Gray (1834) and Heude (1890). Comparisons of our Liuzhou populations against these historical characterizations therefore constitute an indirect line of evidence. Nonetheless, Liu et al. [42] provides the most comprehensive framework currently available for morphological discrimination of Chinese *Cipangopaludina*, under which the two species are readily diagnosed by their shell proportions and whorl profiles [30,42]. Traditional morphometrics thus validated these classical traits quantitatively, but it could not capture fine whorl surface details or aperture curvature.

Discrete landmark-based geometric morphometrics (24 landmarks) afforded higher-resolution shell shape characterization. Thin-plate spline deformation grids localized interspecific divergence to three key regions, namely the outer aperture lip and the first whorl flank adjacent to the aperture (Figure 2c,d). These deformation patterns align precisely with established diagnostic traits. *Cipangopaludina cathayensis*_lz exhibits an expanded aperture and inflated adjacent whorl consistent with its broadly conical-ovoid form and convex whorl surfaces, as well as a depressed apex matching its short, broad spire [31]. By contrast, *C. chinensis*_lz exhibits a retracted aperture and elongated apex corresponding to its slender, high-spired outline. This landmark-based framework reliably captures holistic whorl and aperture geometry. Analogous approaches have been widely adopted to delimit closely related viviparid species [2,56] and detect intraspecific sexual shape dimorphism within the family [31,33]. Nevertheless, limited landmark density precludes resolution of subtle variation in the fine apex curvature.

The high-density semilandmark approach with 200 equidistant points outperformed discrete landmarks in both dimensionality reduction efficiency and fine-scale shape resolution. The first three principal components accounted for nearly all total shape variation, indicating far higher information efficiency per component relative to the discrete landmark dataset [57,58]. This method not only reproduced all major deformation patterns identified by discrete landmarks but also resolved additional subtle shape differences including lateral whorl contraction or expansion and fine-scale apex morphological variation (Figure 2e,f). These fine-scale features align directly with documented morphological disparities between the two species. *Cipangopaludina chinensis* exhibits coordinated rapid growth in both whorl height and width, while *C. cathayensis* shows preferential width expansion and strongly convex whorl surfaces [42]. These patterns were only partially captured by discrete landmark analysis. This approach further demonstrated that the characteristically inflated, stout body whorl of *C. cathayensis* extends across the lateral whorl surface, a morphological detail not emphasized in prior taxonomic descriptions. The improved resolution of subtle shell traits via high-density semilandmarks is consistent with findings from other gastropod morphometric studies [17]. For morphological structures with limited unambiguous homologous landmarks, quantitative comparisons have shown semilandmark-based analyses outperform traditional landmark methods in detecting subtle shape variation [58,59]. Both geometric morphometric approaches reliably discriminate the two species. The high-density semilandmark method captures more shape variation with fewer components and resolves subtle shell features beyond the resolution of discrete landmarks (Figure 2a,b). Core diagnostic traits include aperture size, first whorl contour, apex morphology, and umbilicus position. No significant sexual dimorphism was detected in *C. cathayensis*_lz, whereas *C. chinensis*_lz showed only weak sex-associated differentiation (Figure 3c, Table 2). The observed shape divergence cannot be attributed to allometric scaling, as all analyses were size-standardized (Procrustes alignment, ratio indices). Moreover, both morphospecies were collected from the shallow littoral zone of the same pond, with no observed microhabitat differences in water depth, substrate type or vegetation affinity; this sympatric co-occurrence in a homogeneous microhabitat indicates that ecophenotypic plasticity alone cannot explain the consistent and pronounced shell differentiation [2,24]. The persistent morphological gap is likely sustained by heritable divergence, potentially driven by reproductive isolation or long-term disruptive selection on functional shell traits [60,61,62]. However, no evidence of fine-scale niche partitioning was recovered from field observations, and ecological divergence therefore remains unsupported by the present dataset. The exact selective mechanisms underlying morphological divergence require further functional ecological investigation.

### 4.2. Molecular Evidence: Mitonuclear Conflict and Limited Genetic Differentiation

In contrast to the clear morphological partition, molecular data exhibit substantial mitonuclear discordance and a more complex phylogenetic pattern. The BEAST2 species tree and mitochondrial gene tree (*COI* + *16S rRNA*) consistently recovered *C. chinensis* and *C. cathayensis* as monophyletic (Figure 4 and Figure 5). While the species-tree backbone is largely robust, the mitonuclear conflict among outgroups manifests as a reversed relative placement of *P. lineatus* and *G. aegis*. The mitochondrial gene tree recovers a branching order consistent with the well-established higher-level molluscan phylogeny, while the nuclear gene tree yields a deviant topology for these two lineages. This anomaly is primarily driven by the low phylogenetic informativeness of the conserved nuclear markers (*28S rRNA* and *H3*) at deep taxonomic scales; the limited number of loci and broad taxonomic span of outgroups further reduce topological robustness at deep nodes [4,63]. Critically, this deviation is restricted entirely to outgroup taxa outside Viviparidae and does not affect our core inferences regarding species boundaries within *Cipangopaludina*, as the focal genus Cipangopaludina and its species are recovered within a well-supported, monophyletic Viviparidae clade (PP = 1.0, Figure 5).

The four markers employed—mitochondrial (*COI*, *16S rRNA*) and nuclear (*H3*, *28S rRNA*)—are standard loci in gastropod molecular systematics and have been widely used in viviparid phylogenetics [1,4,10,34,64,65]. Mitochondrial markers recovered all seven nominal species as monophyletic with high support, confirming their utility at the interspecific level. In contrast, the nuclear markers are more conserved and better suited for resolving deeper phylogenetic relationships than for discriminating recently diverged lineages [66,67,68], as reflected in their substantially lower genetic diversity compared to the mitochondrial markers (Table S3). This limited informativeness at shallow divergence scales may therefore contribute to the paraphyly of *C. chinensis*_lz and *C. cathayensis*_lz in the nuclear gene tree. Distinguishing between insufficient marker resolution and genuine biological processes (e.g., incomplete lineage sorting or introgression) will require genome-wide data, which we identify as a priority for future work.

The nuclear gene tree (*H3* + *28S rRNA*) failed to recover reciprocal monophyly between *C. chinensis*_lz and *C. cathayensis*_lz, instead showing them intermixed with *C. wisseli*, *C. chinensis* and *C. cathayensis* in paraphyletic or polytomous arrangements (Figure 5). This pronounced mitonuclear discordance—strong mitochondrial differentiation contrasted with unresolved nuclear topology—constitutes the primary molecular incongruence of our study. Such discordance is not uncommon in molluscan systematics, as exemplified by Bivalvia, where mitochondrial markers recover Amarsipobranchia whereas nuclear markers support Heteroconchia [64]; the cone snail *Lautoconus ventricosus* (Gmelin, 1791) shows similar patterns with incomplete lineage sorting and introgression [69]; within freshwater gastropods, phylogenomic analyses of East Asian viviparids resolved four mitochondrial clades but only two nuclear clusters [65]. These precedents demonstrate that mitonuclear incongruence is pervasive across Mollusca, underscoring the value of integrating multiple genetic datasets for robust species delimitation. Furthermore, the genetic diversity patterns confirm that mitochondrial markers carry the primary phylogeographic signal, while nuclear markers provide little resolution at the population level, consistent with the observed mitonuclear discordance.

We acknowledge that the nuclear markers employed (*H3* and *28S rRNA*) have limited resolving power for fine-scale population-level relationships. However, the mitonuclear conflicts observed here are not confined to the focal populations—they also involve deep phylogenetic nodes among major gastropod lineages (Figure 5). This pattern of conflicting, yet well-supported, topologies at both shallow and deep taxonomic levels suggests that the discordance reflects genuine evolutionary processes (e.g., incomplete lineage sorting or introgression) rather than insufficient marker resolution.

The haplotype networks provide additional insights. In both *COI* and *16S rRNA*, *C. chinensis*_lz and *C. cathayensis*_lz shared haplotypes (Figure 6), suggesting shared ancestry or limited gene flow. Crucially, neither population shared haplotypes with GenBank sequences of *C. chinensis* or *C. cathayensis*. Two non-exclusive explanations could account for this pattern. First, the Liuzhou populations may represent a distinct, previously unsampled lineage within the *C. chinensis* complex. Second, the lack of haplotype sharing may reflect limitations in available GenBank data—reference sequences could be misidentified or derive from geographically distant, sparsely sampled populations that inadequately represent the genetic diversity of these nominal species [12]. Current data do not allow discrimination between these alternatives. Nevertheless, the consistent morphological separation and sympatry of *C. chinensis*_lz and *C. cathayensis*_lz support their recognition as distinct morphospecies, irrespective of haplotype interpretation. This finding aligns with the growing awareness that many nominal viviparid species exhibit mitonuclear conflicts or genetic homogeneity despite morphological divergence [2,10].

### 4.3. Reconciling the Discordance: Species Validity Despite Genetic Mixing

Given the strong morphological separation but weak and conflicting genetic differentiation, the preponderance of evidence supports recognizing *C. chinensis*_lz and *C. cathayensis*_lz as two valid morphospecies, while acknowledging that their genetic boundaries are not fully resolved.

Firstly, under a morphological species concept, the shell divergence between the two taxa is clear and consistent. This is evidenced by complete non-overlap in multivariate spaces, Mahalanobis distances exceeding 10 (Table 1), and sympatric co-occurrence. Such divergence far exceeds typical intraspecific variation documented in viviparids [2,30,65]. Furthermore, in the absence of evidence for ecophenotypic plasticity—since both populations were collected from the same habitat—the observed morphological gap most likely reflects either reproductive isolation or long-term disruptive selection maintaining discrete phenotypic clusters [24,70]. Additionally, a non-mutually exclusive possibility is that *C. chinensis*_lz and *C. cathayensis*_lz represent ecotypes or morphs maintained by frequency-dependent or balancing selection [68,71], which could generate and maintain discrete phenotypic variation even in the absence of complete reproductive isolation. Distinguishing among these hypotheses would require population genomic scans for signatures of selection, and behavioral or ecological studies to evaluate potential fitness trade-offs.

Secondly, the lack of reciprocal monophyly in nuclear genes and the shared haplotypes in mitochondrial genes do not necessarily invalidate species status. It is well documented in freshwater gastropods that morphologically distinct nominal species can be genetically indistinguishable or show mitonuclear discordance [2,10,23,36,72]. Such discordance can arise from incomplete lineage sorting (ILS)—the retention of ancestral polymorphisms following speciation—particularly when effective population sizes are large and divergence is relatively recent [71]. This phenomenon is not restricted to shallow timescales; in Mollusca, ancient ILS has been shown to persist even at deep phylogenetic nodes (~520 Ma) when rapid diversification outpaces coalescent sorting [71], reinforcing ILS as a pervasive source of phylogenetic discordance across evolutionary timescales [73,74,75]. The observed nuclear mixing may therefore reflect ILS, although limited introgression cannot be excluded. Importantly, the complete absence of haplotype sharing between the Liuzhou populations and GenBank sequences of *C. chinensis* indicates that *C. chinensis*_lz and *C. cathayensis*_lz are not misidentified but represent a distinct genetic cluster, further supporting their specific distinctiveness.

Thirdly, the observed differences in phylogenetic signals between mitochondrial and nuclear markers do not themselves argue against species validity; rather, they reflect the different evolutionary dynamics of these two genomic compartments. In Mollusca, numerous well-documented cases illustrate that such discordance commonly coexists with valid species boundaries. For instance, mitochondrial markers recover the monophyly of Amarsipobranchia (Heterodonta + Pteriomorphia), whereas nuclear markers support the monophyly of Heteroconchia (Heterodonta + Palaeoheterodonta), yet both major clades represent well-established higher-level taxa [64]. In the *Octopus vulgaris* (Cuvier, 1797) species complex, genome-wide SNP data revealed significant mitonuclear discordance. Nuclear loci supported *O. vulgaris* sensu stricto and Type III (South Africa) as distinct species, a relationship not recovered by mtDNA [76]. Despite this discordance, both lineages remain recognized as valid taxonomic units. Similarly, the Mediterranean cone snail *Lautoconus ventricosus* exhibits pronounced mitonuclear discordance with instances of incomplete lineage sorting and introgression; species delimitation tests nonetheless proposed the existence of at least three valid species [69]. In freshwater gastropods, the New Zealand snail *Potamopyrgus antipodarum* (Gray, 1843) shows strikingly distinct nuclear versus mitochondrial genomic signatures, yet it is recognized as a cohesive species [77]. While these examples differ from our system in divergence depth and marker resolution, they demonstrate that mitonuclear discordance can persist across timescales without undermining species validity. Notably, discordance is recovered even with higher-resolution genomic data. Collectively, these molluscan studies support the broader principle that mitonuclear conflict does not preclude valid species recognition.

While our sample size (31 individuals per species) is comparable to that of many integrative taxonomic studies in freshwater gastropods, we recognize that increased sampling across multiple populations and localities would further strengthen the generality of our conclusions. The present findings should therefore be considered as a foundation for broader geographic sampling in future studies.

Given the discordance between morphological and molecular evidence, we weighted morphological data more heavily in our taxonomic interpretation based on five criteria: (1) the exceptionally strong and consistent morphological separation (complete non-overlap, Mahalanobis distances > 10, and all traits significant at *p* < 0.001); (2) the taxonomic relevance of the shell characters used [30,42]; (3) the low variability and limited resolving power of the nuclear markers (*H3* and *28S rRNA*) for recent divergences [75,77]; (4) sympatric sampling, which reduces the likelihood of ecophenotypic plasticity [24]; and (5) a conservative management approach that prioritizes safeguarding potentially adaptive phenotypic diversity. These criteria jointly justify placing greater interpretive weight on morphology in this specific case.

Therefore, we conclude that the morphological evidence, combined with the sympatric occurrence and the genetic distinctness from GenBank references, strongly supports treating *C. chinensis*_lz and *C. cathayensis*_lz as two valid species, while acknowledging that their genetic boundaries are not fully closed.

### 4.4. Implications for Taxonomy and Conservation

Our results underscore the necessity of integrative taxonomy when morphology and molecules conflict. Relying solely on morphology risks over-splitting by misinterpreting genetic mixing as intraspecific variation, whereas genetics alone risks under-splitting by failing to detect species boundaries masked by incomplete lineage sorting. For the *C. chinensis*–*C. cathayensis* complex, we recommend retaining both as valid morphospecies, while recognizing the Liuzhou populations (designated *C. chinensis*_lz and *C. cathayensis*_lz) as distinct evolutionarily significant units (ESUs) that do not fully align with GenBank reference sequences from topotypical populations. The geographic suffix “_lz” explicitly distinguishes our sympatric study populations from published conspecific sequences, consistent with the absence of mitochondrial haplotype sharing. This notation preserves the morphological correspondence to the two classical species, while acknowledging their genetic distinctiveness as a previously unsampled lineage within the *C. chinensis* complex, and avoids overstating their taxonomic status prior to genome-level validation. This conclusion reinforces the view that species delimitation in viviparids requires integrative evidence combining morphology, multi-locus genetics, and, where feasible, genomic data [4,6,9].

Future work should employ population whole-genome resequencing to resolve nuclear admixture and clarify the relationship between the Liuzhou populations and topotypical material. Whole-genome data provide unbiased, genome-wide variant discovery, avoiding the ascertainment limitations of reduced-representation approaches [4]. With the available chromosome-level genome of *C. cathayensis* [13] and declining sequencing costs [9], this approach is now feasible for non-model organisms. It would enable robust tests of genetic clustering, gene flow, and selection signatures potentially linked to morphological divergence. From a conservation standpoint, both taxa are harvested and play important ecological roles in freshwater ecosystems [13,15]. We therefore recommend managing *C. chinensis*_lz and *C. cathayensis*_lz as separate units pending further genomic evidence, to safeguard their genetic diversity and local adaptations. Specific actions should include: (i) independent population monitoring; (ii) avoiding stock intermixing without genetic screening; and (iii) genome-wide validation to inform regulatory status. If genomic data confirm limited gene flow, conservation efforts should be strengthened; if connectivity is high, management may be relaxed. In either case, the precautionary separation is justified by current evidence of morphological distinctiveness and evolutionary significance.

## 5. Conclusions

This integrative study reveals a pronounced discordance between morphology and molecules in the two sympatric Chinese river snails, *C. chinensis*_lz and *C. cathayensis*_lz. Shell shape completely separates the two taxa, whereas molecular data show low level differentiation with mitonuclear conflicts. Despite nuclear mixing and haplotype sharing, the absence of haplotype sharing with GenBank references, combined with clear morphometric separation and sympatric occurrence, supports recognizing them as valid species. The mitonuclear discordance likely reflects incomplete lineage sorting or historical introgression, but genome-wide data are needed to resolve these processes. We conclude that *C. chinensis*_lz and *C. cathayensis*_lz are valid species, though their genetic boundaries remain partially porous. This study underscores the value of integrative taxonomy for species delimitation in morphologically challenging freshwater gastropods. From a conservation perspective, the two species should be managed as separate units to preserve their genetic diversity and local adaptations.

## Figures and Tables

**Table 1:** Interspecific morphological distances and Goodall’s F-tests between *C. chinensis*_lz and *C. cathayensis*_lz. Lower-triangular values (*C. chinensis*_lz vs. *C. cathayensis*_lz) are based on the 24-landmark geometric morphometric dataset, and upper-triangular values (*C. cathayensis*_lz vs. *C. chinensis*_lz) are based on the 200-point high-density semilandmark dataset. Mahalanobis and Procrustes distances are given together with Goodall’s *F*-statistics; all permutation tests (10,000 iterations) were significant at *p* < 0.0001.

	Mahalanobis Distances		Procrustes Distances		Goodall’s *F* Tests	
	*C. chinensis*_lz	*C. cathayensis*_lz	*C. chinensis*_lz	*C. cathayensis*_lz	*C. chinensis*_lz	*C. cathayensis*_lz
*C. chinensis*_lz		12.3682		0.0524		106.1928
*C. cathayensis*_lz	15.4901		0.0573		37.0984	

**Table 2:** Pairwise Mahalanobis and Procrustes distances among the four species-sex groups (ZGC, ZGX, ZHC, ZHX). Lower-triangular values are derived from the 24-landmark geometric morphometric dataset, and upper-triangular values from the 200-point high-density semilandmark dataset. All permutation tests (10,000 iterations) were significant at *p* < 0.0001, except for comparisons involving same-species sexes (see text for details).

	Mahalanobis Distances				Procrustes Distances			
	ZGC	ZGX	ZHC	ZHX	ZGC	ZGX	ZHC	ZHX
ZGC		3.8391	12.0114	12.3953		0.0119	0.0514	0.0451
ZGX	25.2567		12.4203	12.7506	0.0139		0.0605	0.0537
ZHC	20.9306	42.0034		4.0049	0.0650	0.0666		0.0087
ZHX	48.0067	71.8304	35.7758		0.0512	0.0529	0.0192	

## Data Availability

The data are contained within the article, and all sequence data are available from the corresponding author upon request (gqh@hunnu.edu.cn). Newly generated *28S rRNA*, *16S rRNA*, *COI*, and *H3* sequences are available in GenBank (accession number: PZ545870-PZ545924, PZ545927-PZ545980, PZ545982-PZ546036, PZ578035-PZ578089).

## Supplementary Materials

The following supporting information can be downloaded at: https://www.mdpi.com/article/10.3390/biology15151317/s1, Figure S1: Positions of 24 landmarks superimposed on a photograph of *Cipangopaludina* (the *C. chinensis*_lz was photographed using a Canon EOS R50 digital camera in this photograph); Table S1: Taxon names, specimen codes (ID), and accession numbers for all sequences used in the phylogenetic analyses. √, means the specimen was used. *n*, number; Table S2: Shell morphometric measurements (mm) of *Cipangopaludina chinensis*_lz and *Cipangopaludina cathayensis*_lz: shell height (SH), shell width (SW), spire height (SpH), body whorl height (BWH), aperture width (AW), operculum height (OH), and operculum width (OW); Table S3: Genetic diversity indices for *C. chinensis*_lz and *C. cathayensis*_lz based on four genes (*COI*, *16S rRNA*, *H3*, and *28S rRNA*); Table S4: Taxon names, ID, and GenBank accession numbers for sequences used in the haplotype network analyses.
