# Supplementary material for: Morphological Divergence Without Genetic Differentiation in Widely Distributed Chinese Cipangopaludina Species
Source: Biology (Basel). 2026 Aug 6;15(15):1317. doi: 10.3390/biology15151317 (PMC13465762; doi:10.3390/biology15151317)
Supplement: Supplementary file 1 [file biology-15-01317-s001.zip › Figure S1.pdf]

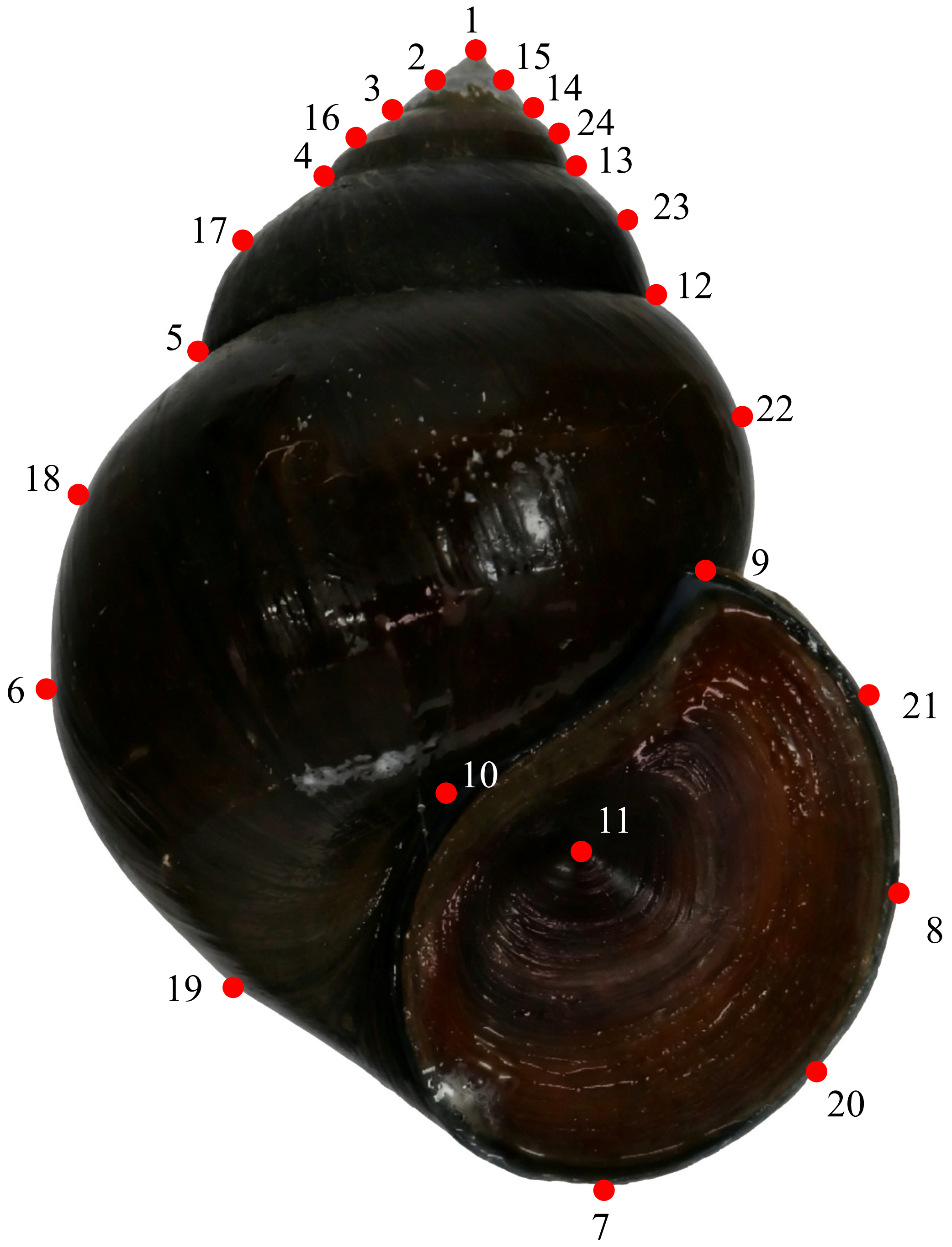

Figure S1. Positions of 24 landmarks superimposed on a photograph of *Cipangopaludina* (the *C. chinensis*_lz was photographed using a Canon EOS R50 digital camera in this photograph)
