# Supplementary material for: Morphological Divergence Without Genetic Differentiation in Widely Distributed Chinese Cipangopaludina Species
Source: Biology (Basel). 2026 Aug 6;15(15):1317. doi: 10.3390/biology15151317 (PMC13465762; doi:10.3390/biology15151317)
Supplement: Supplementary file 1 [file biology-15-01317-s001.zip › Table S1-S4.pdf]

Table S1 Taxon names, specimen codes (ID), and accession numbers for all sequences used in the phylogenetic analyses. √, means the specimen was used. *n*, number

**Species**	**ID**	**Accession number**				**Species tree** $n = 32$	**Gene tree** $n = 81$
		16S rRNA	*28S rRNA*	*COI*	*H3*		
Steromphala cineraria	*Steromphala cineraria*	AY340458.1	AY340395.1	KU496638.1	KX277600.1	√	√
Phorcus lineatus	*Phorcus lineatus*	KX277380.1	U82422.1	KU566799.1	KX277611.1	√	√
Chrysomallon squamiferum	*Chrysomallon squamiferum*	AY163398.1	KP898446.1	MN840613.1	KP898447.1	√	√
Gigantopelta aegis	*Gigantopelta aegis*	KX966246.1	KX966248.1	KR024405.1	KX966249.1	√	√
Gibbula magus	*Gibbula magus*	KX277400.1	AY145406.1	GQ232364.1	KX277621.1	√	√
Viviparus ater	*Viviparus ater*	FJ405734.1	FJ405630.1	FJ405882.1	FJ405774.1	√	√
Viviparus contectus	*Viviparus contectus*	FJ405692.1	FJ405634.1	FJ405835.1	FJ405737.1	√	√
Provanna sp.	*Provanna* sp. 1	GQ290480.1	GQ290518.1	KY581540.1	GQ290620.1	√	√
Provanna sp.	*Provanna* sp. 2	GQ290479.1	GQ290517.1	GQ290578.1	GQ290619.1	√	√
Cipangopaludina chinensis	*Cipangopaludina chinensis* V18	LC028476.1	LC505917.1	LC028540.1	LC505961.1	√	√
	Cipangopaludina chinensis V817	LC437688.1	LC505916.1	LC437752.1	LC505960.1	√	√
	Cipangopaludina chinensis V766	LC437685.1	LC505915.1	LC437749.1	LC505959.1	√	√
	Cipangopaludina chinensis NCU57HN1	PX631134	PX570099	PX631129			√
	Cipangopaludina chinensis NCU1FJ9	PX631135	PX570100	PX631130			√
	Cipangopaludina chinensis NCU35YT4	PX631136	PX570101	PX631131			√
Cipangopaludina japonica	*Cipangopaludina japonica* V311	LC028465.1	LC505921.1	LC028529.1	LC505965.1	√	√
	Cipangopaludina japonica V254	LC028463.1	LC505920.1	LC028527.1	LC505964.1	√	√
	Cipangopaludina japonica Cip jap 1	FJ405736.1	FJ405621.1	FJ405876.1	FJ405777.1	√	√
Cipangopaludina zejaensis	*Cipangopaludina zejaensis* V715	LC505860.1	LC505946.1	LC505800.1	LC505983.1	√	√
	Cipangopaludina zejaensis V710	LC505859.1	LC505945.1	LC505799.1	LC505982.1	√	√
Cipangopaludina sujfunensis	*Cipangopaludina sujfunensis* V714	LC505858.1	LC505944.1	LC505798.1	LC505981.1	√	√

**Species**	**ID**	*16S rRNA*	*28S rRNA*	*COI*	*H3*	**Species tree**	**Gene tree**
Cipangopaludina ussuriensis	*Cipangopaludina ussuriensis* V1471	LC505857.1	LC505943.1	LC505797.1	LC505980.1	√	√
	Cipangopaludina ussuriensis V717	LC505856.1	LC505942.1	LC505796.1	LC505979.1	√	√
Cipangopaludina wisseli	*Cipangopaludina wisseli* ZMB127112a		MN999597	MN997927.1	MN997755.1		√
Cipangopaludina compactus	*Cipangopaludina compactus* NCU65LP14	PX631132	PX570097	PX631127		√	√
Cipangopaludina cathayensis	*Cipangopaludina catayensis* NCU74ML2	PX631133	PX570098	PX631128		√	√
Cipangopaludina chinensis	*Cipangopaludina chinensis*_lz01c	PZ545927	PZ545870	PZ545982	PZ578035		√
	*Cipangopaludina chinensis*_lz02c	PZ545928	PZ545871	PZ545983	PZ578036		√
	*Cipangopaludina chinensis*_lz03c	PZ545929	PZ545872	PZ545984	PZ578037		√
	*Cipangopaludina chinensis*_lz05c	PZ545930	PZ545873	PZ545985	PZ578038		√
	*Cipangopaludina chinensis*_lz06c	PZ545931	PZ545874	PZ545986	PZ578039		√
	*Cipangopaludina chinensis*_lz07c	PZ545932	PZ545875	PZ545987	PZ578040		√
	*Cipangopaludina chinensis*_lz08c	PZ545933	PZ545876	PZ545988	PZ578041		√
	*Cipangopaludina chinensis*_lz09c	PZ545934	PZ545877	PZ545989	PZ578042		√
	*Cipangopaludina chinensis*_lz10c	PZ545935	PZ545878	PZ545990	PZ578043		√
	*Cipangopaludina chinensis*_lz11c	PZ545936	PZ545879	PZ545991	PZ578044		√
	*Cipangopaludina chinensis*_lz12c	PZ545937	PZ545880	PZ545992	PZ578045		√
	*Cipangopaludina chinensis*_lz13c	PZ545938	PZ545881	PZ545993	PZ578046		√
	*Cipangopaludina chinensis*_lz14c	PZ545939	PZ545882	PZ545994	PZ578047		√
	*Cipangopaludina chinensis*_lz15c	PZ545940	PZ545883	PZ545995	PZ578048		√
Cipangopaludina chinensis	*Cipangopaludina chinensis*_lz01x	PZ545941	PZ545884	PZ545996	PZ578049		√
	*Cipangopaludina chinensis*_lz02x	PZ545942	PZ545885	PZ545997	PZ578050		√
	*Cipangopaludina chinensis*_lz03x	PZ545943	PZ545886	PZ545998	PZ578051		√
	*Cipangopaludina chinensis*_lz04x	PZ545944	PZ545887	PZ545999	PZ578052		√
	*Cipangopaludina chinensis*_lz05x	PZ545945	PZ545888	PZ546000	PZ578053		√

	ID	*16S rRNA*	*28S rRNA*	*COI*	*H3*	**Species tree**	**Gene tree**
	*Cipangopaludina chinensis*_lz06x	PZ545946	PZ545889	PZ546001	PZ578054	√	√
	*Cipangopaludina chinensis*_lz07x	PZ545947	PZ545890	PZ546002	PZ578055	√	√
	*Cipangopaludina chinensis*_lz08x	PZ545948	PZ545891	PZ546003	PZ578056	√	√
	*Cipangopaludina chinensis*_lz09x	PZ545949	PZ545892	PZ546004	PZ578057	√	√
	*Cipangopaludina chinensis*_lz10x	PZ545950	PZ545893	PZ546005	PZ578058	√	√
	*Cipangopaludina chinensis*_lz11x	PZ545951	PZ545894	PZ546006	PZ578059		√
	*Cipangopaludina chinensis*_lz12x	PZ545952	PZ545895	PZ546007	PZ578060		√
	*Cipangopaludina chinensis*_lz13x	PZ545953	PZ545896	PZ546008	PZ578061		√
	*Cipangopaludina chinensis*_lz14x	PZ545954	PZ545897	PZ546009	PZ578062		√
	*Cipangopaludina chinensis*_lz15x	PZ545955	PZ545898	PZ546010	PZ578063		√
Cipangopaludina cathayensis	*Cipangopaludina cathayensis*_lz02c	PZ545956	PZ545899	PZ546011	PZ578064		√
	*Cipangopaludina cathayensis*_lz03c		PZ545900	PZ546012	PZ578065		√
	*Cipangopaludina cathayensis*_lz04c	PZ545957	PZ545901	PZ546013	PZ578066		√
	*Cipangopaludina cathayensis*_lz05c	PZ545958	PZ545902	PZ546014	PZ578067		√
	*Cipangopaludina cathayensis*_lz06c	PZ545959	PZ545903	PZ546015	PZ578068		√
	*Cipangopaludina cathayensis*_lz07c	PZ545960	PZ545904	PZ546016	PZ578069		√
	*Cipangopaludina cathayensis*_lz08c	PZ545961	PZ545905	PZ546017	PZ578070		√
	*Cipangopaludina cathayensis*_lz10c	PZ545962	PZ545906	PZ546018	PZ578071		√
	*Cipangopaludina cathayensis*_lz11c	PZ545963	PZ545907	PZ546019	PZ578072		√
	*Cipangopaludina cathayensis*_lz12c	PZ545964	PZ545908	PZ546020	PZ578073		√
	*Cipangopaludina cathayensis*_lz13c	PZ545965	PZ545909	PZ546021	PZ578074		√
	*Cipangopaludina cathayensis*_lz14c	PZ545966	PZ545910	PZ546022	PZ578075		√
	*Cipangopaludina cathayensis*_lz15c	PZ545967	PZ545911	PZ546023	PZ578076		√
Cipangopaludina cathayensis	*Cipangopaludina cathayensis*_lz02x	PZ545968	PZ545912	PZ546024	PZ578077		√

	ID	*16S rRNA*	*28S rRNA*	*COI*	*H3*	Species tree	Gene tree
	*Cipangopaludina cathayensis*_lz03x	PZ545969	PZ545913	PZ546025	PZ578078		√
	*Cipangopaludina cathayensis*_lz04x	PZ545970	PZ545914	PZ546026	PZ578079		√
	*Cipangopaludina cathayensis*_lz05x	PZ545971	PZ545915	PZ546027	PZ578080		√
	*Cipangopaludina cathayensis*_lz06x	PZ545972	PZ545916	PZ546028	PZ578081	√	√
	*Cipangopaludina cathayensis*_lz07x	PZ545973	PZ545917	PZ546029	PZ578082	√	√
	*Cipangopaludina cathayensis*_lz08x	PZ545974	PZ545918	PZ546030	PZ578083	√	√
	*Cipangopaludina cathayensis*_lz09x	PZ545975	PZ545919	PZ546031	PZ578084	√	√
	*Cipangopaludina cathayensis*_lz11x	PZ545976	PZ545920	PZ546032	PZ578085	√	√
	*Cipangopaludina cathayensis*_lz12x	PZ545977	PZ545921	PZ546033	PZ578086		√
	*Cipangopaludina cathayensis*_lz13x	PZ545978	PZ545922	PZ546034	PZ578087		√
	*Cipangopaludina cathayensis*_lz14x	PZ545979	PZ545923	PZ546035	PZ578088		√
	*Cipangopaludina cathayensis*_lz15x	PZ545980	PZ545924	PZ546036	PZ578089		√

Table S2 Shell morphometric measurements (mm) of *Cipangopaludina chinensis*_lz and *Cipangopaludina cathayensis*_lz: shell height (SH), shell width (SW), spire height (SpH), body whorl height (BWH), aperture width (AW), operculum height (OH), and operculum width (OW).

Samples	SH	SW	SpH	BWH	AW	OH	OW
*Cipangopaludina chinensis*_lz01x	58.08	36.96	31.41	38.25	32.62	29.54	23.35
*Cipangopaludina chinensis*_lz02x	58.41	35.63	31.84	38.46	32.22	28.46	20.71
*Cipangopaludina chinensis*_lz03x	56.14	35.52	30.94	36.79	30.84	27.37	19.75
*Cipangopaludina chinensis*_lz04x	55.54	34.50	30.53	39.12	31.40	27.78	20.86
*Cipangopaludina chinensis*_lz05x	56.64	35.85	31.51	36.39	32.17	28.66	21.21
*Cipangopaludina chinensis*_lz06x	60.88	37.63	33.69	41.05	33.77	29.49	21.61
*Cipangopaludina chinensis*_lz07x	52.51	32.21	27.83	35.20	29.43	26.71	19.47
*Cipangopaludina chinensis*_lz08x	52.98	32.49	29.27	36.26	29.53	26.74	19.02
*Cipangopaludina chinensis*_lz09x	58.21	36.13	32.16	38.61	32.48	29.89	20.91
*Cipangopaludina chinensis*_lz10x	59.21	36.72	32.75	39.95	33.14	30.12	22.42
*Cipangopaludina chinensis*_lz11x	56.64	36.44	31.06	38.28	31.66	28.44	21.89
*Cipangopaludina chinensis*_lz12x	60.17	36.95	33.29	40.15	33.80	30.42	21.76
*Cipangopaludina chinensis*_lz13x	54.47	33.78	29.89	35.38	30.16	26.99	20.06
*Cipangopaludina chinensis*_lz14x	53.04	33.26	27.81	35.19	30.47	26.33	20.09
*Cipangopaludina chinensis*_lz15x	54.41	33.50	29.09	35.87	30.19	27.18	19.56
*Cipangopaludina chinensis*_lz16x	56.65	34.31	30.90	37.90	31.17	27.67	21.30
*Cipangopaludina chinensis*_lz01c	51.84	34.41	28.59	34.75	29.05	25.60	19.87
*Cipangopaludina chinensis*_lz02c	54.88	34.66	30.88	34.66	30.57	27.20	18.87
*Cipangopaludina chinensis*_lz03c	58.93	36.01	32.25	36.71	32.60	28.33	19.92
*Cipangopaludina chinensis*_lz04c	57.24	36.40	32.32	37.47	31.73	28.25	19.51
*Cipangopaludina chinensis*_lz05c	53.79	34.11	29.38	36.81	29.90	27.26	18.98
*Cipangopaludina chinensis*_lz06c	55.78	36.08	31.20	38.99	30.87	27.45	18.79
*Cipangopaludina chinensis*_lz07c	54.68	34.91	29.43	38.60	30.40	27.61	19.82
*Cipangopaludina chinensis*_lz08c	57.01	35.69	32.09	39.21	32.07	27.23	18.47
*Cipangopaludina chinensis*_lz09c	55.48	35.24	31.44	39.01	30.65	26.91	19.18
*Cipangopaludina chinensis*_lz10c	55.96	35.01	32.21	37.62	30.92	27.88	20.24
*Cipangopaludina chinensis*_lz11c	54.04	34.40	29.33	37.02	30.11	26.85	20.24
*Cipangopaludina chinensis*_lz12c	53.79	34.64	29.74	36.79	28.35	25.89	20.49
*Cipangopaludina chinensis*_lz13c	55.41	34.82	30.44	36.62	29.94	27.75	19.86
*Cipangopaludina chinensis*_lz14c	52.50	34.21	28.52	37.65	28.97	26.70	20.05
*Cipangopaludina chinensis*_lz15c	55.18	35.33	30.49	37.07	30.41	27.36	19.85
*Cipangopaludina cathayensis*_lz01x	55.13	34.55	27.61	39.09	33.63	30.27	21.27
*Cipangopaludina cathayensis*_lz02x	55.07	35.39	28.68	39.30	33.65	29.63	21.17
*Cipangopaludina cathayensis*_lz03x	45.77	30.78	23.79	31.68	28.31	26.37	18.90
*Cipangopaludina cathayensis*_lz04x	44.99	30.65	22.65	33.81	29.18	26.05	19.35
*Cipangopaludina cathayensis*_lz05x	45.96	30.36	24.11	33.30	27.63	24.16	17.25
*Cipangopaludina cathayensis*_lz06x	54.48	34.52	28.50	38.35	31.88	28.17	20.36
*Cipangopaludina cathayensis*_lz07x	50.01	31.28	25.85	33.88	29.08	26.36	18.01

Samples	SH	SW	SpH	BWH	AW	OH	OW
*Cipangopaludina cathayensis*_lz08x	47.67	32.96	25.21	34.11	29.61	26.87	19.42
*Cipangopaludina cathayensis*_lz09x	49.87	33.08	27.04	33.55	30.14	27.33	20.39
*Cipangopaludina cathayensis*_lz10x	44.08	30.41	23.03	31.49	27.80	24.71	18.92
*Cipangopaludina cathayensis*_lz11x	52.58	34.03	26.77	36.27	31.53	27.96	20.47
*Cipangopaludina cathayensis*_lz12x	46.62	32.20	24.76	33.51	28.90	25.85	17.50
*Cipangopaludina cathayensis*_lz13x	52.96	34.64	26.41	38.79	32.81	29.11	20.12
*Cipangopaludina cathayensis*_lz14x	53.58	35.61	27.18	38.73	32.73	29.49	20.42
*Cipangopaludina cathayensis*_lz15x	48.68	31.56	25.44	33.15	29.36	26.44	18.90
*Cipangopaludina cathayensis*_lz16x	45.72	30.65	24.70	32.35	28.38	25.89	18.25
*Cipangopaludina cathayensis*_lz01c	52.35	34.46	27.67	36.42	32.56	28.89	20.11
*Cipangopaludina cathayensis*_lz02c	48.37	31.94	24.84	35.89	30.47	27.13	18.20
*Cipangopaludina cathayensis*_lz03c	53.99	36.77	28.84	38.74	32.34	28.43	21.79
*Cipangopaludina cathayensis*_lz04c	45.84	31.04	24.41	34.21	27.67	24.33	17.73
*Cipangopaludina cathayensis*_lz05c	51.94	35.23	27.36	36.29	31.45	28.20	19.89
*Cipangopaludina cathayensis*_lz06c	51.78	34.88	27.26	35.41	31.66	26.97	20.19
*Cipangopaludina cathayensis*_lz07c	52.14	35.71	26.54	38.33	31.42	29.68	20.71
*Cipangopaludina cathayensis*_lz08c	49.42	33.37	25.22	35.70	29.43	27.11	20.11
*Cipangopaludina cathayensis*_lz09c	49.74	32.59	26.07	35.29	29.98	26.98	19.19
*Cipangopaludina cathayensis*_lz10c	53.61	35.23	28.50	37.97	31.69	28.31	20.13
*Cipangopaludina cathayensis*_lz11c	45.60	31.61	23.25	35.16	28.81	25.36	17.10
*Cipangopaludina cathayensis*_lz12c	43.06	29.66	23.32	30.42	27.50	25.01	15.93
*Cipangopaludina cathayensis*_lz13c	58.21	37.81	30.32	43.82	34.89	31.51	22.91
*Cipangopaludina cathayensis*_lz14c	48.97	32.90	25.70	34.59	30.06	26.33	19.17
*Cipangopaludina cathayensis*_lz15c	46.76	31.44	23.62	35.94	29.61	25.91	18.89

Table 3. Genetic diversity indices for *C. chinensis*_lz and *C. cathayensis*_lz based on four Genes (*COI*, *16S rRNA*, *H3*, and *28S rRNA*).

Genes (length bp)		Populations	n	S	H	Hd	π
		Combined	55	46	22	0.912 ± 0.022	0.0134 ± 0.0019
COI	789	*C. chinensis*_lz	29	38	17	0.933 ± 0.029	0.0139 ± 0.0025
		*C. cathayensis*_lz	26	11	11	0.880 ± 0.045	0.0130 ± 0.0029
		Combined	54	15	6	0.656 ± 0.039	0.0076 ± 0.0016
16S rRNA	438	*C. chinensis*_lz	29	14	5	0.672 ± 0.059	0.0081 ± 0.0022
		*C. cathayensis*_lz	25	12	4	0.657 ± 0.055	0.0072 ± 0.0023
		Combined	55	3	5	0.492 ± 0.071	0.0024 ± 0.0004
H3	272	*C. chinensis*_lz	29	3	5	0.608 ± 0.007	0.0030 ± 0.0006
		*C. cathayensis*_lz	26	2	3	0.342 ± 0.110	0.0017 ± 0.0006
		Combined	55	0	1	0.000 ± 0.000	0.0000 ± 0.0000
28S rRNA	315	*C. chinensis*_lz	29	0	1	0.000 ± 0.000	0.0000 ± 0.0000
		*C. cathayensis*_lz	26	0	1	0.000 ± 0.000	0.0000 ± 0.0000

n, number of individuals; S, number of polymorphic sites; H, number of haplotypes; Hd, haplotype diversity; π, nucleotide diversity. Combined indicates pooled data from both populations.

Table S4 Taxon names, ID, and GenBank accession numbers for sequences used in the haplotype network analyses.

Species name	ID	*16S rRNA*, *n* =235	*COI*, *n* =244
Cipangopaludina zejaensis	C_zejaensis_V715	LC505860.1	LC505800.1
	C_zejaensis_V710	LC505859.1	LC505799.1
Cipangopaludina sujfunensis	C_sujfunensis_V714	LC505858.1	LC505798.1
Cipangopaludina ussuriensis	C_ussuriensis_V1471	LC505857.1	LC505797.1
	C_ussuriensis_UGSB23019		MN997924.1
	C_ussuriensis_UGSB23018		MN997923.1
	C_ussuriensis_V717	LC505856.1	LC505796.1
Cipangopaludina japonica	C_japonica_V981	LC505837.1	
	C_japonica_V980	LC505836.1	
	C_japonica_V920	LC505835.1	
	C_japonica_V919	LC505834.1	
	C_japonica_V994	LC505833.1	LC505777.1
	C_japonica_V993	LC505832.1	LC505776.1
	C_japonica_V1142	LC505831.1	LC505775.1
	C_japonica_V1139	LC505830.1	LC505774.1
	C_japonica_V594	LC505829.1	LC505773.1
	C_japonica_V667	LC505828.1	LC505772.1
	C_japonica_V1072	LC505827.1	LC505771.1
	C_japonica_V1071	LC505826.1	LC505770.1
	C_japonica_V504	LC505825.1	LC505769.1
	C_japonica_V503	LC505824.1	LC505768.1
	C_japonica_V467	LC505823.1	LC505767.1
	C_japonica_V466	LC505822.1	LC505766.1
	C_japonica_V455	LC505821.1	LC505765.1
	C_japonica_V1036	LC505820.1	LC505764.1
	C_japonica_V1035	LC505819.1	LC505763.1
	C_japonica_V1114	LC505818.1	LC505762.1
	C_japonica_V1126	LC505817.1	LC505761.1
	C_japonica_V1017	LC505816.1	LC505760.1
	C_japonica_V1016	LC505815.1	LC505759.1
	C_japonica_V578	LC505814.1	LC505758.1
	C_japonica_V577	LC505813.1	LC505757.1
	C_japonica_V891	LC505811.1	LC505755.1
	C_japonica_V1055	LC505812.1	LC505756.1
	C_japonica_V1128	LC505810.1	LC505754.1
	C_japonica_V409	LC028471.1	LC028535.1
	C_japonica_V407	LC028470.1	LC028534.1
	C_japonica_V401	LC028469.1	LC028533.1

	C_japonica_V362	LC028468.1	LC028532.1
	C_japonica_V345	LC028467.1	LC028531.1
	C_japonica_V342	LC028466.1	LC028530.1
Cipangopaludina japonica	C_japonica_V309	LC028464.1	LC028528.1
	C_japonica_V311	LC028465.1	LC028529.1
	C_japonica_V254	LC028463.1	LC028527.1
	C_japonica_V252	LC028462.1	
	C_japonica_V39	LC028461.1	LC028525.1
	C_japonica_Cipjap1	FJ405736.1	FJ405876.1
	C_japonica_SERCINVERT1793		MK308308.1
	C_japonica_SERCINVERT1794		MK308324.1
	C_japonica_SERCINVERT1774		MK308281.1
	C_japonica_2		MK053824.1
	C_japonica_1		MK053823.1
	C_japonica_V309		LC028528.1
	C_japonica_V362		LC028524.1
	C_japonica_USNM1296945		MN997926.1
Cipangopaludina chinensis	C_chinensis_V1311	LC437748.1	LC437809.1
	C_chinensis_V1310	LC437747.1	LC437808.1
	C_chinensis_V1265	LC437746.1	LC437807.1
	C_chinensis_V1263	LC437745.1	LC437805.1
	C_chinensis_V1251	LC437744.1	LC437804.1
	C_chinensis_V1250	LC437743.1	LC437803.1
	C_chinensis_V1223	LC437742.1	LC437802.1
	C_chinensis_V1222	LC437741.1	LC437801.1
	C_chinensis_V1208	LC437740.1	LC437800.1
	C_chinensis_V1206	LC437739.1	
	C_chinensis_V1205	LC437738.1	
	C_chinensis_V1204	LC437737.1	
	C_chinensis_V1199	LC437736.1	
	C_chinensis_V1155	LC437735.1	LC437798.1
	C_chinensis_V1154	LC437734.1	LC437797.1
	C_chinensis_V1087	LC437733.1	LC437796.1
	C_chinensis_V1086	LC437732.1	LC437795.1
	C_chinensis_V1085	LC437731.1	LC437794.1
	C_chinensis_V1084	LC437730.1	LC437793.1
	C_chinensis_V1065	LC437729.1	LC437792.1
	C_chinensis_V1064	LC437728.1	LC437791.1
	C_chinensis_V1046	LC437727.1	LC437790.1
	C_chinensis_V1045	LC437726.1	LC437789.1
	C_chinensis_V1031	LC437725.1	LC437788.1
	C_chinensis_V1030	LC437724.1	LC437787.1

	C_chinensis_V1029	LC437723.1	LC437786.1
	C_chinensis_V984	LC437722.1	LC437785.1
	C_chinensis_V983	LC437721.1	LC437784.1
	C_chinensis_V856	LC437720.1	LC437783.1
Cipangopaludina chinensis	C_chinensis_V855	LC437719.1	LC437782.1
	C_chinensis_V841	LC437718.1	LC437781.1
	C_chinensis_V840	LC437717.1	LC437780.1
	C_chinensis_V756	LC437716.1	LC437779.1
	C_chinensis_V747	LC437715.1	LC437777.1
	C_chinensis_V746	LC437714.1	LC437776.1
	C_chinensis_V745	LC437713.1	LC437775.1
	C_chinensis_V737	LC437712.1	LC437774.1
	C_chinensis_V736	LC437711.1	LC437773.1
	C_chinensis_V735	LC437710.1	LC437772.1
	C_chinensis_V705	LC437709.1	LC437771.1
	C_chinensis_V656	LC437708.1	LC437769.1
	C_chinensis_V654	LC437707.1	
	C_chinensis_V653	LC437706.1	LC437768.1
	C_chinensis_V632	LC437705.1	LC437767.1
	C_chinensis_V553	LC437704.1	LC437766.1
	C_chinensis_V532	LC437703.1	LC437765.1
	C_chinensis_V531	LC437702.1	LC437764.1
	C_chinensis_V530	LC437701.1	LC437763.1
	C_chinensis_V481	LC437700.1	LC437762.1
	C_chinensis_V173	LC437699.1	
	C_chinensis_V172	LC437698.1	
	C_chinensis_V1368	LC437697.1	LC437761.1
	C_chinensis_V1174	LC437696.1	LC437760.1
	C_chinensis_V1173	LC437695.1	LC437759.1
	C_chinensis_V854	LC437694.1	LC437758.1
	C_chinensis_V853	LC437693.1	LC437757.1
	C_chinensis_V852	LC437692.1	LC437756.1
	C_chinensis_V851	LC437691.1	LC437755.1
	C_chinensis_V850	LC437690.1	LC437754.1
	C_chinensis_V818	LC437689.1	LC437753.1
	C_chinensis_V817	LC437688.1	LC437752.1
	C_chinensis_V768	LC437687.1	LC437751.1
	C_chinensis_V767	LC437686.1	LC437750.1
	C_chinensis_V766	LC437685.1	LC437749.1
	C_chinensis_BHV1	KY426911.1	
	C_chinensis_BGF4	KY426910.1	
	C_chinensis_V344	LC028484.1	LC028548.1

	C_chinensis_V410	LC028483.1	LC028547.1
	C_chinensis_V393	LC028482.1	LC028546.1
	C_chinensis_V384	LC028481.1	LC028545.1
	C_chinensis_V365	LC028480.1	LC028544.1
	C_chinensis_V343	LC028479.1	LC028543.1
Cipangopaludina chinensis	C_chinensis_V249	LC028478.1	LC028542.1
	C_chinensis_V215	LC028477.1	LC028541.1
	C_chinensis_V18	LC028476.1	LC028540.1
	C_chinensis_V170	LC028475.1	LC028539.2
	C_chinensis_V15	LC028474.1	LC028538.2
	C_chinensis_M22436	LC028473.1	LC028537.2
	C_chinensis_V32	LC028472.1	LC028536.2
	C_chinensis_HI91	FJ710214.1	FJ710298.1
	C_chinensis_KR31	FJ710213.1	EU528474.1
	C_chinensis_V1264		LC437806.1
	C_chinensis_V1207		LC437799.1
	C_chinensis_V755		LC437778.1
	C_chinensis_V657		LC437770.1
	C_chinensis_2		MK053826.1
	C_chinensis_1		MK053825.1
	C_chinensis_BIOUG13626H10		MG421641.1
	C_chinensis_BIOUG13626G04		MG421204.1
	C_chinensis_BIOUG13626G11		MG421748.1
	C_chinensis_NCU_YY18	PX614418	PX568279
	C_chinensis_NCU_HA	PX614419	
	C_chinensis_NCU_LP9	PX614420	PX568280
	C_chinensis_NCU_YC1		PX568281
	C_chinensis_NCU_HN10	PX614421	PX568282
	C_chinensis_NCU_J16x	PX614422	PX568283
	C_chinensis_NCU_YT2	PX614423	PX568284
	C_chinensis_NCU_FJ15	PX614424	PX568285
	C_chinensis_NCU_Hehu	PX614425	
	C_chinensis_ZMB192694		MN997925.1
	C_chinensis_NCU57HN1	PX631134	PX631129
	C_chinensis_NCU1FJ9	PX631135	PX631130
	C_chinensis_NCU35YT4	PX631136	PX631131
Cipangopaludina sp.	C_sp_KMMU149	KP009573.1	KP009560.1
	C_sp_KMMU152	KP009572.1	KP009566.1
	C_sp_KIZD179	GU198873.1	GU198819.1
	C_sp_KIZD68	GU198845.1	GU198791.1
	C_sp_KIZD67	GU198844.1	GU198790.1
	C_sp_KIZD66	GU198843.1	GU198789.1

	C_sp_KIZD65	GU198842.1	GU198788.1
	C_sp_KIZD63	GU198841.1	GU198787.1
	C_sp_KIZD62	GU198840.1	GU198786.1
	C_sp_KIZD60	GU198838.1	GU198784.1
	C_sp_KIZD59	GU198837.1	GU198783.1
Cipangopaludina ventricosa	C_ventricosa_Cv	KJ867107.1	
Cipangopaludina longispira	C_longispira_Cl	KJ867106.1	
	C_longispira_KIZD149	GU198864.1	GU198810.1
	C_longispira_KIZD148	GU198863.1	GU198809.1
Cipangopaludina dianchiensis	C_dianchiensis_KIZD178	GU198872.1	GU198818.1
	C_dianchiensis_KIZD177	GU198871.1	
	C_dianchiensis_KIZD61	GU198839.1	GU198785.1
	C_dianchiensis_KIZD44	GU198836.1	GU198782.1
	C_dianchiensis_KIZD43	GU198835.1	GU198781.1
	C_dianchiensis_GZ01		JN621284.1
Cipangopaludina cf.	C_cf_TT617		LC901182.1
	C_cf_TT499		LC901181.1
	C_cf_TT498		LC901180.1
	C_cf_TT497		LC901179.1
Cipangopaludina malleata	C_malleata_ DNAS58E8CSX		ON229904.1
Cipangopaludina compactus	C_compactus_NCU_LGHS1	PX614391	
	C_compactus_NCU_EYTX	PX614392	PX568253
	C_compactus_NCU_EHT	PX614393	
	C_compactus_NCU_JC1	PX614394	PX568254
	C_compactus_NCU_JC7	PX614395	PX568255
	C_compactus_NCU_ML6	PX614396	PX568256
	C_compactus_NCU_YY13	PX614397	PX568257
	C_compactus_NCU_ZHS1	PX614398	PX568258
	C_compactus_NCU_SC3	PX614399	PX568259
	C_compactus_NCU_KY	PX614400	PX568260
	C_compactus_NCU_LZ9	PX614401	PX568261
	C_compactus_NCU_NN14	PX614402	PX568262
	C_compactus_NCU_LT	PX614403	PX568263
	C_compactus_NCU_YF4	PX614404	PX568264
	C_compactus_NCU_HB11	PX614405	PX568265
	C_compactus_NCU_HN13	PX614406	PX568266
	C_compactus_NCU_JX1	PX614407	PX568267
	C_compactus_NCU65LP14	PX631132	PX631127
Cipangopaludina catayensis	C_catayensis_NCU_ML74	PX614408	PX568268
	C_catayensis_NCU_LP2	PX614409	PX568269
	C_catayensis_NCU_SD6	PX614410	PX568270
	C_catayensis_NCU74ML2	PX631133	PX631128

Cipangopaludina chinensis fluminalis	C_chinensis_fluminalis_NCU_WX7	PX614411	PX568271
	C_chinensis_fluminalis_NCU_W10x	PX614412	PX568272
	C_chinensis_fluminalis_NCU_W12x	PX614413	PX568273
Cipangopaludina wisseli	C_wisseli_ZMB127112a		MN997927
Cipangopaludina latissima	C_latissima_NCUKuanTi01		PX568278
Cipangopaludina chinensis	*Cipangopaludina chinensis*_lz01c	PZ545927	PZ545982
	*Cipangopaludina chinensis*_lz02c	PZ545928	PZ545983
	*Cipangopaludina chinensis*_lz03c	PZ545929	PZ545984
	*Cipangopaludina chinensis*_lz05c	PZ545930	PZ545985
	*Cipangopaludina chinensis*_lz06c	PZ545931	PZ545986
	*Cipangopaludina chinensis*_lz07c	PZ545932	PZ545987
	*Cipangopaludina chinensis*_lz08c	PZ545933	PZ545988
	*Cipangopaludina chinensis*_lz09c	PZ545934	PZ545989
	*Cipangopaludina chinensis*_lz10c	PZ545935	PZ545990
	*Cipangopaludina chinensis*_lz11c	PZ545936	PZ545991
	*Cipangopaludina chinensis*_lz12c	PZ545937	PZ545992
	*Cipangopaludina chinensis*_lz13c	PZ545938	PZ545993
	*Cipangopaludina chinensis*_lz14c	PZ545939	PZ545994
	*Cipangopaludina chinensis*_lz15c	PZ545940	PZ545995
	*Cipangopaludina chinensis*_lz01x	PZ545941	PZ545996
	*Cipangopaludina chinensis*_lz02x	PZ545942	PZ545997
	*Cipangopaludina chinensis*_lz03x	PZ545943	PZ545998
	*Cipangopaludina chinensis*_lz04x	PZ545944	PZ545999
	*Cipangopaludina chinensis*_lz05x	PZ545945	PZ546000
	*Cipangopaludina chinensis*_lz06x	PZ545946	PZ546001
	*Cipangopaludina chinensis*_lz07x	PZ545947	PZ546002
	*Cipangopaludina chinensis*_lz08x	PZ545948	PZ546003
	*Cipangopaludina chinensis*_lz09x	PZ545949	PZ546004
	*Cipangopaludina chinensis*_lz10x	PZ545950	PZ546005
	*Cipangopaludina chinensis*_lz11x	PZ545951	PZ546006
	*Cipangopaludina chinensis*_lz12x	PZ545952	PZ546007
	*Cipangopaludina chinensis*_lz13x	PZ545953	PZ546008
	*Cipangopaludina chinensis*_lz14x	PZ545954	PZ546009
	*Cipangopaludina chinensis*_lz15x	PZ545955	PZ546010
Cipangopaludina cathayensis	*Cipangopaludina cathayensis*_lz03c		PZ546012
	*Cipangopaludina cathayensis*_lz04c	PZ545957	PZ546013
	*Cipangopaludina cathayensis*_lz05c	PZ545958	PZ546014
	*Cipangopaludina cathayensis*_lz06c	PZ545959	PZ546015
	*Cipangopaludina cathayensis*_lz07c	PZ545960	PZ546016
	*Cipangopaludina cathayensis*_lz08c	PZ545961	PZ546017

	*Cipangopaludina cathayensis*_lz10c	PZ545962	PZ546018
	*Cipangopaludina cathayensis*_lz11c	PZ545963	PZ546019
	*Cipangopaludina cathayensis*_lz12c	PZ545964	PZ546020
	*Cipangopaludina cathayensis*_lz13c	PZ545965	PZ546021
Cipangopaludina cathayensis	*Cipangopaludina cathayensis*_lz14c	PZ545966	PZ546022
	*Cipangopaludina cathayensis*_lz15c	PZ545967	PZ546023
	*Cipangopaludina cathayensis*_lz02x	PZ545968	PZ546024
	*Cipangopaludina cathayensis*_lz03x	PZ545969	PZ546025
	*Cipangopaludina cathayensis*_lz04x	PZ545970	PZ546026
	*Cipangopaludina cathayensis*_lz05x	PZ545971	PZ546027
	*Cipangopaludina cathayensis*_lz06x	PZ545972	PZ546028
	*Cipangopaludina cathayensis*_lz07x	PZ545973	PZ546029
	*Cipangopaludina cathayensis*_lz08x	PZ545974	PZ546030
	*Cipangopaludina cathayensis*_lz09x	PZ545975	PZ546031
	*Cipangopaludina cathayensis*_lz11x	PZ545976	PZ546032
	*Cipangopaludina cathayensis*_lz12x	PZ545977	PZ546033
	*Cipangopaludina cathayensis*_lz13x	PZ545978	PZ546034
	*Cipangopaludina cathayensis*_lz14x	PZ545979	PZ546035
	*Cipangopaludina cathayensis*_lz15x	PZ545980	PZ546036
